# The Natural Cytotoxicity Receptors in Health and Disease

**DOI:** 10.3389/fimmu.2019.00909

**Published:** 2019-05-07

**Authors:** Alexander David Barrow, Claudia Jane Martin, Marco Colonna

**Affiliations:** ^1^Department of Microbiology and Immunology, Peter Doherty Institute for Infection and Immunity, University of Melbourne, Melbourne, VIC, Australia; ^2^Department of Pathology and Immunology, Washington University School of Medicine, St. Louis, MO, United States

**Keywords:** receptors, natural killer cell, immunoregulation, disease association, tissue homeostasis

## Abstract

The Natural Cytotoxicity Receptors (NCRs), NKp46, NKp44, and NKp30, were some of the first human activating Natural Killer (NK) cell receptors involved in the non-MHC-restricted recognition of tumor cells to be cloned over 20 years ago. Since this time many host- and pathogen-encoded ligands have been proposed to bind the NCRs and regulate the cytotoxic and cytokine-secreting functions of tissue NK cells. This diverse set of NCR ligands can manifest on the surface of tumor or virus-infected cells or can be secreted extracellularly, suggesting a remarkable NCR polyfunctionality that regulates the activity of NK cells in different tissue compartments during steady state or inflammation. Moreover, the NCRs can also be expressed by other innate and adaptive immune cell subsets under certain tissue conditions potentially conferring NK recognition programs to these cells. Here we review NCR biology in health and disease with particular reference to how this important class of receptors regulates the functions of tissue NK cells as well as confer NK cell recognition patterns to other innate and adaptive lymphocyte subsets. Finally, we highlight how NCR biology is being harnessed for novel therapeutic interventions particularly for enhanced tumor surveillance.

## Introduction

Natural Killer (NK) cells constitute a population of large, granular lymphocytes that are located in the blood, lymphoid organs, such as the thymus and spleen, and non-lymphoid organs, such as the liver and uterus, as well as tissues, such as skin (1–4). NK cells were originally identified based on their ability to lyse certain tumor and virally infected cells (5, 6). In contrast, normal healthy cells that express sufficient levels of MHC class I molecules are spared from NK cell attack (7). Subsequently, the process whereby the effector functions of developing NK cells are adapted to the levels of MHC class I expressed by a host, termed NK cell “education”, was described (8, 9). It was during this exciting era of discovery that the functional activity of NK cells was shown to be exquisitely controlled by inhibitory receptors specific for MHC class I molecules, such as the Ly49 receptors in mice (10), and the Killer immunoglobulin-like receptors (KIR) in humans (11–14). Fully educated NK cells efficiently lyse target cells lacking MHC class I molecules, implying the existence of a set of activating NK cell receptors for non-MHC class I ligands expressed on target cells that are either not present or expressed at much lower levels on healthy cells, although formal molecular evidence for this was lacking during this period.

In contrast to T and B lymphocytes, NK cells lack the expression of rearranging cell-surface antigen receptors and so it remained unclear how NK cells might be aroused by surface ligands expressed by tumor or virus-infected cells. Even though NK cells lack expression of the T cell receptor (TCR), they still retain expression of the ζ chain from the CD3 signaling complex. These data suggested that NK cells express cell-surface receptors that might share similar downstream signaling mechanisms to the TCR but are regulated by different ligands. For example, whereas T cells recognize short antigenic peptides in the context of MHC class I, NK cells can be inhibited by the expression of MHC class I molecules. Thus, NK cells express activating receptors that may recognize ligands expressed by tumor and virus-infected cells in a non-MHC-restricted fashion. Whilst the function and downstream signaling events of the low affinity Fc receptor, CD16, that mediates antibody-dependent cellular cytotoxicity (ADCC) were well-defined at this time (15–17), the identity of other activating receptors and their cognate ligands involved in the natural cytotoxicity of NK-susceptible targets cells were largely unknown, which stimulated further research into the receptors that trigger NK cell cytotoxicity.

The Natural cytotoxicity receptors (NCRs) were originally identified by the Moretta group almost 20 years ago in a series of elegant redirected lysis experiments using human NK cells (18–20). The NCR family are comprised of three type I transmembrane (TM) receptors, termed NKp46, NKp44, and NKp30, which are encoded by the genes, *NCR1, NCR2*, and *NCR3*, respectively. Even though the NCRs were discovered based on their ability to induce NK cell cytotoxicity of monoclonal antibody (mAb)-coated tumor cell targets, the blocking of individual NCR activity using soluble mAbs had only a mild effect on NK cell cytotoxicity and different tumor cells varied in their susceptibility. Combinations of soluble mAbs to the NCRs were found to have a much stronger blocking effect for selected tumor cell-lines indicating that the NCRs can cooperate with each other to mediate NK cell cytotoxicity of certain tumor cell-types (18, 20, 21). These results suggest that the coordinated expression of NCR ligands by different tumor cell-types as well as the level of NCR expression by different NK cell clones governs NK cell cytotoxicity, which is counterbalanced by the level of MHC class I expressed for a given tumor cell type.

Recent success in identifying the various ligands for the NCRs is now beginning to illuminate the biological roles that this important class of receptors plays in NK cell surveillance of malignant or pathogen-infected cells and in different tissue microenvironments. One striking aspect that has become apparent is that each NCR may interact with several different pathogen- and host-encoded molecules that can either be expressed on cell-surfaces, secreted or shed extracellularly, or incorporated into the extracellular matrix (ECM). Moreover, cytokines expressed in the local tissue microenvironment can influence which particular NCR isoform is expressed. These different NCR isoforms have now been shown to deliver either activating or inhibitory signaling functions depending on their interaction with ligand. Here we review NCR biology in the different tissue NK cell populations as well as other innate and adaptive immune subsets, their functional interactions with a diverse set of cellular- and pathogen-encoded ligands, and the potential for these interactions to be harnessed for tumor immunotherapy.

### NKp46

**Figure 1:**
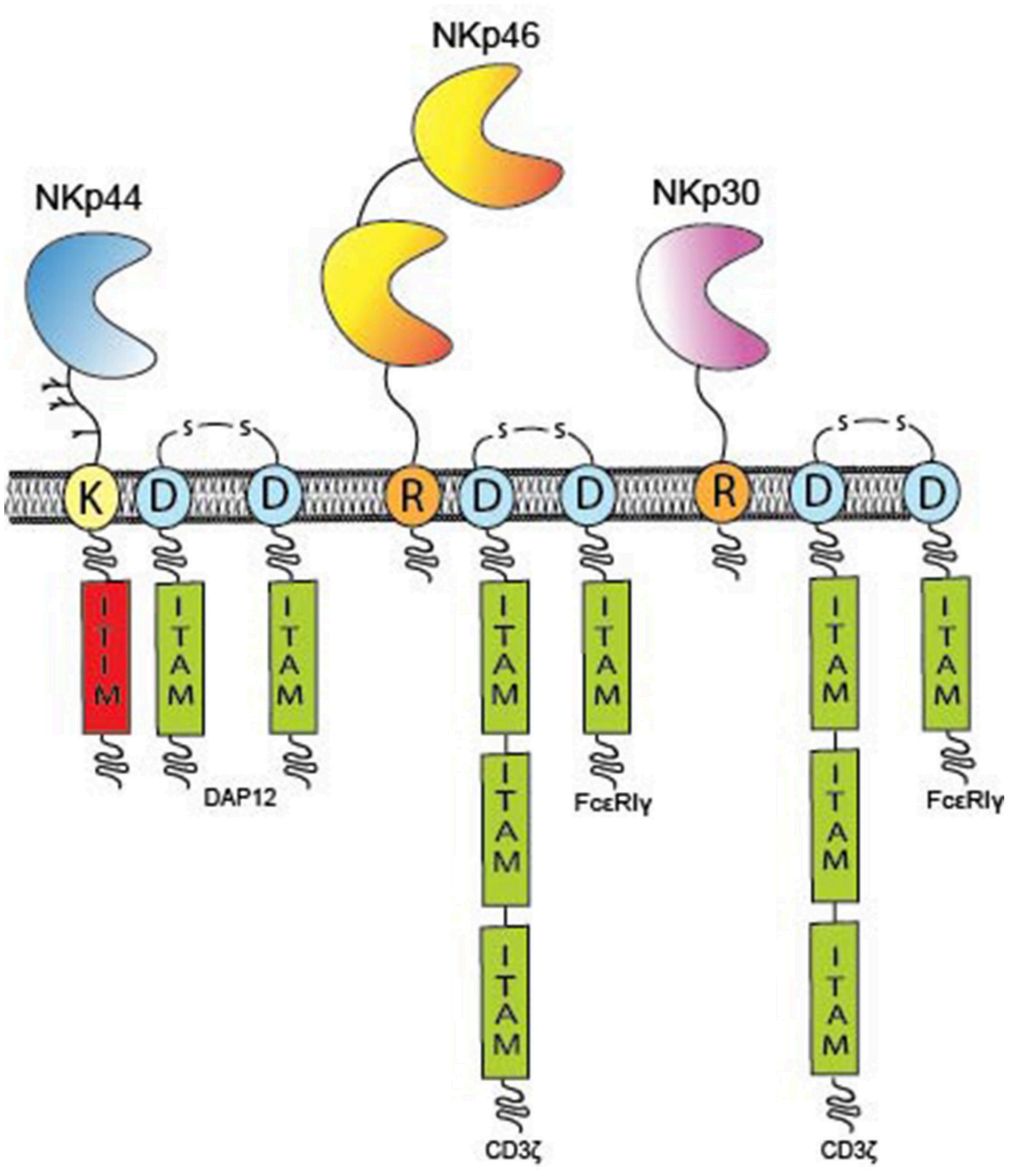
Overview of individual NCR domain structures. The domain architecture of the NCRs and TM signaling adaptors encoding ITAM residues (green boxes) are displayed. The NCRs are type I TM proteins expressed on the plasma membrane of immune cells. NKp46 (yellow) has two Ig-like domains, whereas NKp30 (pink) and NKp44 (blue) possess only one Ig-like domain. All NCRs contain either a positively charge arginine (R) or lysine (K) residue in their hydrophobic TM domains that can form a salt bridge with a corresponding aspartate (D) residue in the TM domains of the ITAM adaptors; CD3, FcR, or DAP12, respectively. The cytoplasmic domains of the NCRs do not encode any inherent signaling capacity with the exception of NKp44 that contains a putative ITIM sequence (red) in its cytoplasmic tail and thus maintains potential for inhibitory signaling.

Molecular cloning of the cDNA for NKp46 (also known as natural cytotoxicity receptor 1, NCR1) revealed an open reading frame (ORF) encoding a 46 kDa type I TM protein belonging to the immunoglobulin (Ig) superfamily characterized by two extracellular C2-type Ig-like domains followed by a stalk region (Figure 1) (21). The two Ig-like domains of NKp46 are arranged in a V-shaped conformation positioned at an angle of 85° to each other similarly to the D1D2 domains of KIRs and Leukocyte Ig-like receptors [LILR, also known as ILTs (22)] that share a distant ancestral evolutionary relationship with NKp46 (23, 24). The cytoplasmic domain of NKp46 lacks an Immunoreceptor Tyrosine-based Activation Motif (ITAM), instead the TM domain contains a positively charged arginine residue that mediates association with the negatively charged aspartate residue in the TM domain of the ITAM signaling adaptors, CD3ζ or the Fc receptor common γ (FcRγ) (Figure 1). The gene encoding NKp46, *NCR1*, is located in the Leukocyte Receptor Complex (LRC) on chromosome 19q13.4 (25). A murine *NCR1* ortholog has also been cloned and maps to mouse chromosome 7, the syntenic region of human chromosome 19 (21).

The expression of NKp46 on NK cells is conserved across all mammalian species (26). In humans, NKp46 is expressed by all CD56^dim^CD16^+^ and CD56^bright^CD16^−^ human NK cells irrespective of their activation status (19). Cross-linking with anti-NKp46 mAb results in calcium release and the secretion of IFN-γ and TNF-α by NK cells and blocking NKp46 signaling with specific mAbs can result in reduced NK cell cytotoxicity of certain tumor cell-lines, although the most potent blocking activity is observed in combination with mAbs to other NCRs (19, 21). Subsequent studies have now shown that NKp46 is also expressed by innate lymphoid cells (ILCs) of group 1 (ILC1) and a subset of group 3 ILCs (NCR^+^ ILC3) (27, 28), γδ T cells (29, 30), a population of oligoclonally expanded intraepithelial (IEL) cytotoxic T lymphocytes (CTL) (31) and a population of IL-15-dependent innate-like IEL lacking surface TCR expression (32) in celiac disease patients, and umbilical cord blood (UCB) T cells cultured in IL-15 (33). NKp46 is also expressed by malignant NK, NKT, and T cell lymphomas (32, 34–36) (Table 1).

**Table 1:** Expression of Natural cytotoxicity receptors and their ligands.

**Receptor**	**Gene**	**Cellular expression**	**Ligands**	**Ligand-induced signal**	**References**
NKp46	*NCR1*	NK cells, ILC1, NCR^+^ ILC3, Small intestine TCRαβ^+^ CD8^+^ IEL, Small intestine TCRαβ^−^ innate-like IEL, TCRγδ^low^CD3^−^, Expanded peripheral blood γδ T cells (Vδ1^+^), NK, NKT and T lymphomas, Cord blood T cells cultured in IL-15	Heparan sulfate (HS) gylcosaminogylcans (GAGs) HA (hemagglutinin) of influenza virus HA of human vaccinia virus HN of avian Newcastle disease virus, Sendai virus and human parainfluenza virus (DBL)-1 α domain of *Plasmodium falciparum* erythrocyte membrane protein (PfEMP1) Vimentin expressed on cells infected with *Mycobacterium tuberculosis* Unidentified ligand expressed by *Fusobacterium nucleatum* Unidentified ligand expressed by pancreatic β-cells *C. glabrata* Epa proteins Complement Factor P (properdin)	Activation Activation Activation Activation Activation Activation Activation Activation Activation Activation	(52) (53) (54) (53, 55–57) (58) (59, 60) (61) (62) (63) (64)
NKp44	*NCR2*	NK cells, ILC1, ILC3, Plasmacytoid dendritic cells, Small intestine TCRαβ^+^ CD8^+^ IEL, Expanded peripheral blood γδ T cells (Vδ1^+^), Cord blood T cells cultured in IL-15	HS GAGs Syndecan-4 (*in cis*) HA of Influenza virus HN of avian Newcastle disease virus, Sendai virus and human parainfluenza virus PDGF-DD Nidogen-1 PCNA NKp44L expressed on tumor cells, bystander CD4^+^ T cell during HIV infection, or cartilage-derived chondrocytes Domain III envelope protein from West Nile and Dengue viruses Unknown ligand(s) on *Mycobacterium tuberculosis, M. bovis, Nocardia farcinica and Pseudomonas aeruginosa*	Activation Inhibition Activation Activation Activation Inhibition Inhibition Activation Activation Unclear	(52, 65) (65) (66) (55–57) (67) (68) (69, 70) (71–73) (74) (75, 76)
NKp30	*NCR3*	NK cells, CD8^+^ T cells, Expanded peripheral blood γδ T cells (Vδ1^+^), ILC2 Cord blood T cells cultured in IL-15	HS GAGs HA of human vaccinia virus pp65, main tegument protein of human cytomegalovirus (DBL)-1 α domain of Plasmodium falciparum erythrocyte membrane protein (PfEMP1) BAT3/BAG6 B7-H6 Galectin-3 β-1,3 glucan	Activation Inhibition Inhibition Activation Activation Activation Inhibition Activation	(52, 77, 78) (54) (79) (58) (80–82) (83, 84) (85) (86, 87)

### NKp44

The functional activity of NK cells against tumor cells deficient in the expression of MHC class I molecules is greatly enhanced by culture in IL-2, suggesting that NK cells upregulate activating receptors for additional non-MHC ligands. Whereas, NKp30 and NKp46 are constitutively expressed by resting NK cells obtained from peripheral blood, the expression of NKp44, also known as natural cytotoxicity receptor 2 (NCR2), is upregulated on NK cells stimulated by IL-2, IL-15 or IL-1β, particularly on the CD56^bright^ subset (20, 37–39). NKp44 is a 44 kDa protein comprised of a single extracellular V-type Ig (IgV) domain followed by a long stalk region and a hydrophobic TM domain containing a charged lysine residue that mediates association with the ITAM adaptor, DAP12 (also known as KARAP and TYROBP) (Figure 1) (40, 41). Structural studies have shown that the IgV domain of NKp44 forms a saddle-shaped dimer with a positively charged groove on one side of the protein (42).

The gene for NKp44 (*NCR2*) is encoded in the human TREM receptor locus at 6p21.1, which is centromeric of the MHC (20, 43) and is also found in several primates species but not in mice (44). Three major transcripts are expressed from *NCR2* (NKp44-1,−2, and−3) that have been investigated in detail. Whereas, NKp44-2 and NKp44-3 are predicted to encode proteins with short cytoplasmic domains with no inherent signaling capacity, NKp44-1 is predicted to encode a protein with a long cytoplasmic tail containing the amino acid sequence “ILYHTV” that conforms to the sequence of an Immunoreceptor Tyrosine-based Inhibition Motif (ITIM). The NKp44-1 isoform thus has the potential for inhibitory as well as activating signaling since this isoform also retains the capacity to associated with DAP12. A molecular characterization of the NKp44-1 ITIM showed that it has the potential to be phosphorylated in the NK92 NK cell-line but could not recruit the phosphotyrosine phosphatases, SHP-1 or SHP-2, or the 5′-inositol phosphatase, SHIP, in order to mediate cellular inhibition (45). Thus, it was concluded that the activating function of NKp44-1 is not influenced by the presence of the cytoplasmic ITIM. Nevertheless, NKp44-1 has the potential for dual signaling functions through the cytoplasmic ITIM and association with the ITAM adaptor, DAP12.

Like the other NCRs, NKp44 has also been shown to be expressed by ILC1 (46, 47), ILC3 (48, 49), γδ T cells (30, 50), oligoclonally expanded IEL CTL in celiac disease patients (31), and UCB T cells cultured in IL-15 (33), in addition to plasmacytoid dendritic cells where NKp44 may be involved in the regulation of type I interferon secretion (51) (Table 1).

### NKp30

NKp30, also known as natural cytotoxicity receptor 3 (NCR3), was identified as a 30 kDa protein that, similarly to NKp46, is expressed on all mature resting and activated NK cells (18). Molecular cloning of the NKp30 cDNA revealed an open reading frame predicted to encode one extracellular IgV domain and a hydrophobic TM domain with a charged arginine residue capable of associating with the ITAM adaptors, CD3ζ and/or FcRγ (Figure 1) (18, 88). The crystal structure of NKp30 reveals some structural similarity to CTLA-4 and PD-1 and NKp30 is thus considered a member of the CD28 family of receptors (83, 89). Both NKp30 and NKp46 have reduced surface expression on adaptive memory NK cells most likely due to the downregulated expression of the FcRγ signaling chain required for the surface expression of these receptors (90, 91).

In humans, *NCR3* is encoded in the class III region of the Major Histocompatibility Complex (MHC) but was found to be a pseudogene in 13 strains of mice with the exception of *Mus caroli* (92). Six alternatively spliced transcripts are transcribed from the NKp30 gene, termed NKp30a-f. Three NKp30 isoforms, NKp30a, NKp30b, and NKp30c, which differ in their cytoplasmic tails due to alternative splicing in exon 4, have been studied in detail. Whilst NKp30a and NKp30b evoke NK cell activation, the NKp30c isoform was shown to elicit secretion of the immunosuppressive cytokine, IL-10, from NK cells (93). NKp30 has also been shown to be expressed by γδ T cells (30), CD8^+^ T cells (94), and UCB T cells cultured in IL-15 (33).

## NCRs and their Ligands in Cancer

**Figure 2:**
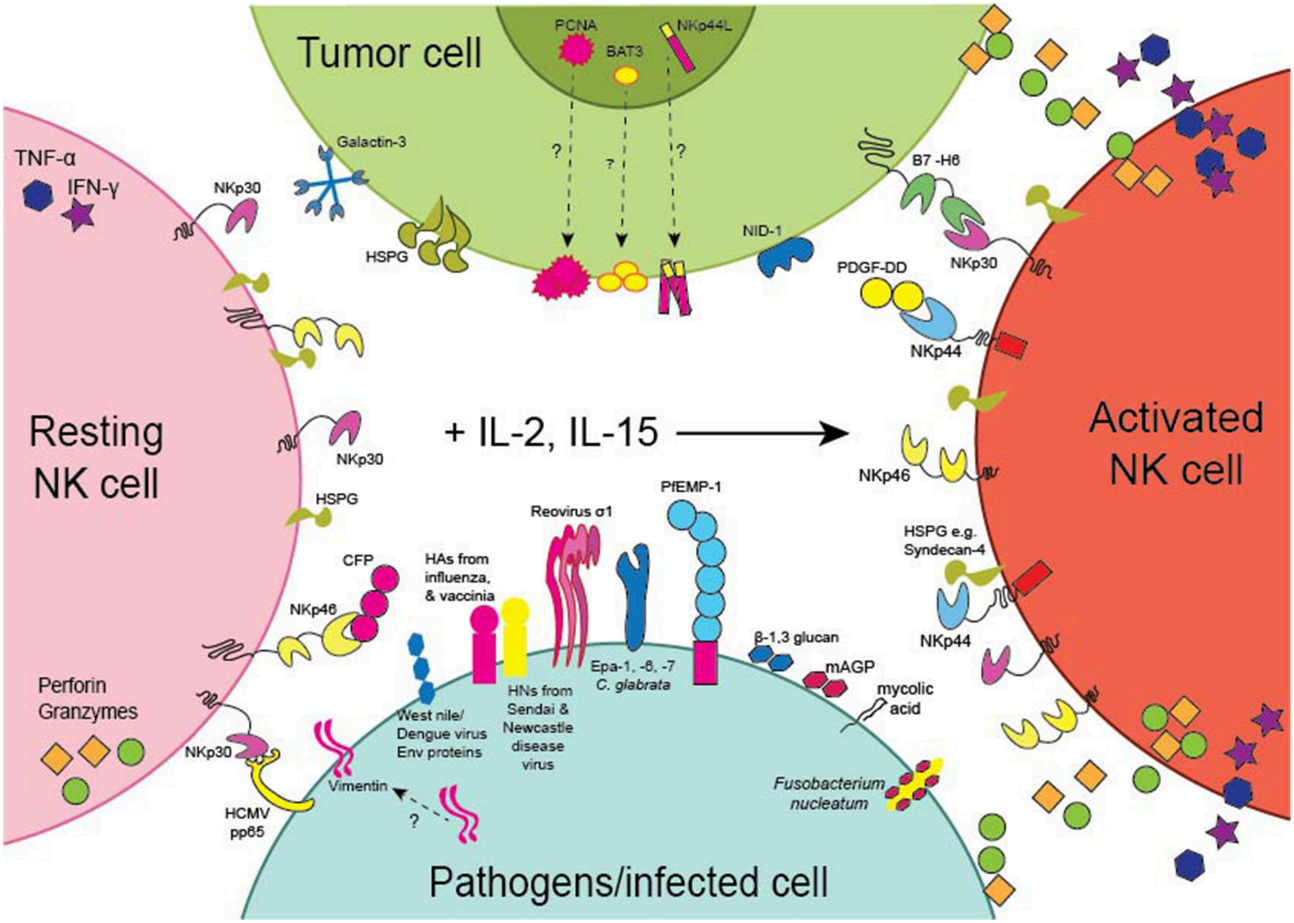
Expression of the NCRs and their Ligands. Schematic representation of the expression of the NCRs on NK cells and the NCR ligands by tumors (green) or pathogens or cells infected with pathogens (light blue). NKp30 and NKp46 are expressed by resting (pink) as well as activated (orange) NK cells, whereas NKp44 is only expressed by NK cells activated with IL-2 or IL-15 (arrow). PCNA, BAT3 and NKp44L are nuclear proteins but the pathways whereby they become expressed on the surface of tumor cells remain to be identified. NKp44L is a splice variant of the Mixed-lineage leukemia 5 (MLL5) protein encoding a C-terminal modification reported to mediate cell-surface expression of NKp44L on tumor cells. Similarly, the pathway whereby vimentin becomes expressed on the surface of *M. tuberculosis* infected cells also remains to be described.

The NCRs were first characterized based on their ability to evoke the cytotoxic and cytokine-secreting functions of NK cells toward tumor cell-lines *in vitro*. Several studies have now provided evidence that the NCRs are involved in tumor surveillance *in vivo*. For example, genetic deficiency of NKp46 in mice results in the impaired clearance of subcutaneous T lymphoma (95) and melanoma (96) tumors and melanoma lung metastases (97, 98). Moreover, transgenic overexpression of NKp46 resulted in the enhanced clearance of melanoma lung metastases (99). Intriguingly, enhanced NKp46 signaling elicited IFN-γ secretion and increased tumor deposition of fibronectin, which altered the solid tumor architecture and resulted in the decreased formation of melanoma metastases (100). Importantly, the T lymphoma and melanoma cell-lines used in these studies all expressed cell-surface ligands for NKp46. Moreover, in human patients with melanoma, normal melanocytes had negligible expression of NKp46 ligands in comparison to malignant melanocytes deep within melanoma lesions that stained strongly for NKp46-Fc showing that NKp46 ligands can be upregulated on malignantly transformed cells but are not expressed by healthy cells (101). Several cellular ligands for the NCRs have now been proposed (Table 1) and we discuss these here in the context of anti-tumor responses (Figure 2).

### Heparan Sulfate Glycosaminoglycans

Heparan sulfate (HS) glycosaminoglycans (GAGs) are found on cell surfaces and within the ECM and consist of long, branched, anionic polysaccharides that are incorporated into proteins to form HS proteoglycans (HSPGs), such as the syndecans and glypicans, that form an integral and dynamic component of normal tissue architecture (102, 103). HSPGs also play vital roles in tumor progression allowing cancer cells to proliferate, elude immunosurveillance, invade neighboring tissues, and metastasize to distal tissue sites from the primary tumor. The negative charge of HSPGs can also provide docking sites for the basic domains of various secreted factors, such as chemokines and growth factors like fibroblast growth factor (104).

All three NCRs have been reported to bind to different HS sequences. HS GAGs are heterogenous in structure with a preference for highly sulfated HS structures and with each NCR possessing a distinct HS binding specificity. NKp30 and NKp46 binding to HS is similar although the binding varies significantly. In contrast, NKp44 displays a very different binding pattern to NKp30 and NKp46 (52, 77). A cis interaction of NKp44 with the cell-associated HSPG, syndecan-4, has been reported. The interaction between syndecan-4 and NKp44 was shown to regulate the membrane distribution of NKp44, and constitutively dampen NKp44 activity. Treatment of NK cells with soluble HS was proposed to disrupt the cis association with syndecan-4 and potentiate NKp44 signaling (65).

It is an attractive hypothesis to speculate that NK cells could utilize the NCRs to sense changes in HSPGs in the tumor microenvironment or possibly even during infection to activate NK cell cytotoxicity and IFN-γ secretion, particularly since several cancers exhibit aberrant regulation of key HS biosynthetic enzymes, such as 3-*O*- and 6-*O*-Sulfotransferases, and catabolic enzymes, such as heparanase and the HS endosulfatases, SULF1 and SULF2 (105). Conversely, pathogens or tumor cells could modify HSPGs to evade NK cell surveillance. Indeed, the over-expression of telomere repeats binding factor 2 (TRF2), a key factor in telomere protection, can result in the upregulation of heparan sulfate-glucosamine 3-*O*-sulfotransferase 4 (HS3ST4) in cancer cells by binding to the interstitial telomeric repeat located within the HS3ST4 intron (106). Silencing of HS3ST4 expression using short hairpin RNAs in cancer cells resulted in increased tumor infiltration of activated NK cells. Thus, aberrant sulfation of cell-surface HSPGs can modify the capacity for tumor surveillance by NK cells. However, the precise role of the NCRs and HS GAGs in NK cell tumor surveillance remains unclear since NKp30-dependent NK cell cytotoxicity was unaffected by GAG-deficiency or heparanase treatment of tumor cell targets (78). It is possible that HS GAGs may also serve as “co-receptors” facilitating interactions with other NCR ligands, such as growth factors like PDGF-DD (67).

### B7-H6

K562 cells are highly susceptible to lysis by human NK cells, which is mediated by NKp30-mediated recognition of a tumor cell-surface ligand. A proteomics approach designed to trap ligands bound to an NKp30 Fc-fusion protein by chemical cross-linking resulted in the co-immunoprecipitation of B7-H6 from K562 cell lysates (84). B7-H6 is type I TM protein possessing two extracellular Ig-like domains and is a member of the B7 family of costimulatory molecules. Like other members of the B7 family, the extracellular domain of B7-H6 is composed of a membrane distal IgV domain and a membrane proximal IgC domain (83). NKp30 was found to use the front and back β-sheets of its IgV domain to engage the side and face of the corresponding β-sandwich of B7-H6, whereas CTLA-4 and PD-1 use only the front β-sheet of their Ig-like domains to contact their ligands. B7-H6 contacts NKp30 through the complementarity-determining region (CDR)-like loops of its IgV domain, thus resembling the binding interaction of antibodies with antigen, which is not observed for CTLA-4 binding to B7.1/B7.2 or PD-1 binding to PD-L1/PD1-L2 (83).

B7-H6 expression was found to be negligible on normal cells but was highly expressed by a wide range of tumor cells, showing that cellular transformation serves as a mode of immunosurveillance in the innate immune system (84). In support of this, the expression of B7-H6 is upregulated by the proto-oncogene Myc (107) and is associated with greater overall survival of patients with oral squamous carcinoma (108). In contrast, some tumors are thought to escape NKp30 recognition by metalloprotease-mediated shedding of B7-H6 from the cell-surface (109). The soluble form of B7-H6 is detected in the serum of patients with hepatocellular carcinoma (110), metastatic gastrointestinal tumors (GIST) (111), neuroblastoma (112, 113), and peritoneal fluid from ovarian cancer patients (114), and is associated with impaired NKp30 expression and NK cell dysfunction as well as poor overall patient survival.

Differential expression of transcripts encoding the different NKp30 isoforms have also been associated with cancer prognosis. The preferential expression of the immunosuppressive NKp30c isoform over the activating NKp30a and NKp30b isoforms was associated with the reduced overall survival of GIST patients treated with imatinib mesylate (93, 111). The NKp30c isoform was reported to trigger secretion of the immunosuppressive cytokine IL-10 from NK cells and was associated with NK cell dysfunction characterized by defective NK cell degranulation and secretion of IFN-γ and TNF-α. Interestingly, polymorphism in the 3′ untranslated region of *NCR3* can result in the preferential expression of the immunosuppressive NKp30c isoform by NK cells and predicted the clinical outcome for GIST patients independently from KIT mutation (93). In pediatric neuroblastoma, expression of the immunostimulatory (NKp30a/b) versus immunosuppressive (NKp30c) isoforms is also associated with a higher risk of relapse and elevated serum levels of soluble B7-H6 that inhibit NK function were associated with the bone marrow metastasis and chemoresistance of neuroblastoma cells (112, 113). Expression of B7-H6 is also detected on monocytes and neutrophils treated with TLR ligands and pro-inflammatory cytokines. A soluble form of B7-H6 was also detected under these conditions and in sepsis patients suggesting B7-H6 may also regulate NK cell activity via NKp30 isoforms during inflammation (115).

### Nuclear Proteins

NKp44 has been reported to bind to several intracellular ligands that have been proposed to be aberrantly expressed on the cell-surface of tumor or virus-infected cells. An NKp44 ligand was reported to be expressed on the surface of tumor or CD4^+^ T cell from HIV patients (71) that was dependent on expression of HIV gp41. Moreover, a peptide sequence derived from HIV gp41 was shown to upregulate the expression of this NKp44 ligand on CD4^+^ T cells (71). A yeast two-hybrid screen using a cDNA library from the Jurkat T cell-line was employed to find interacting partners for NKp44 (72) and a cDNA encoding a splice variant of the mixed-lineage leukemia protein-5 (MLL5) was subsequently cloned. MLL5 is normally a nuclear antigen but the cDNA sequence revealed a new exon resulting in a transcript encoding an MLL5 variant protein with an alternative C-terminal amino-acid sequence. This new protein termed NKp44L was reported to be expressed on the cell-surface and in the cytoplasm of various tumor cell-lines but not in normal tissues (72). However, the mechanism driving NKp44L cell-surface expression remains obscure. NKp44L expression has also been reported on human articular chondrocytes (73).

NKp44 was also reported to interact with Proliferating cell nuclear antigen (PCNA). PCNA is found in the nucleus and functions as a co-factor for DNA polymerase δ that helps leading strand synthesis during DNA replication (116). PCNA was identified as an NKp44 ligand following a screen of a yeast surface display library using an NKp44-Fc fusion protein (69). Cell-surface expression of PCNA was reported following transfection into HeLa cells, which reduced NK cell cytotoxicity and IFN-γ secretion. PCNA was found to localize at the plasma membrane where it was shown to form an immunological synapse with NKp44 to mediate NK cell inhibition. The inhibition of NK cell signaling was reported to occur via the NKp44-1 splice variant that potentially encodes a cytoplasmic ITIM (69). The interaction of PCNA with NKp44 was proposed to form a unique interaction capable of transducing an inhibitory rather than an activating signal (69, 117). PCNA has also been shown to associate with Human Leukocyte Antigen (HLA) class I molecules on the cell-surface of tumor cells to form an inhibitory ligand for NKp44 and suppression of NK cell cytotoxicity (70). The authors suggested that NCR ligands could act as Damage-Associated Molecular Patterns (DAMPs) through association with HLA class I molecules (118). Interestingly, preferential expression of the transcript encoding the ITIM-bearing NKp44-1 isoform is associated with poor survival in acute myeloid leukemia and was proposed to be a novel target for checkpoint blockade on NK cells (119).

HLA-B-associated transcript 3 (BAT3), also known as Bcl2-associated anthogene 6 (BAG6), is another intracellular protein that was identified as an NKp30 ligand using a yeast two-hybrid screening approach (80). BAT3 is a multifunctional molecular chaperone that contributes to several cell processes including apoptosis, gene regulation, protein synthesis, protein quality control and protein degradation. For example, BAT3 can function by preventing the aggregation of misfolded and hydrophobic-patch containing proteins ensuring their correct delivery to the endoplasmic reticulum or the sorting of proteins that have mislocalized to the cytoplasm for proteosomal degradation (120). BAT3 also accumulates in the nucleus where it is involved in regulating apoptosis following DNA damage (121). BAT3 was proposed to be released from tumor cells to engage NKp30 and trigger NK cell activation (80, 81). Exosomal release of BAT3 was also reported to promote NK:DC cross-talk (82). In contrast, the release of a soluble form of BAT3 found in the plasma of CLL patients was found to suppress NK cell activation through competition for the exosomal form of BAT3 and other tumor ligands (122, 123).

### Platelet-Derived Growth Factor-DD

Growth factors (GFs) are important for guiding various cellular and developmental processes and GF pathways are often dysregulated in cancer. The platelet-derived growth factor (PDGF) family are comprised of four polypeptides that can assemble into at least five dimeric isoforms, PDGF-AA, PDGF-BB, PDGF-AB, PDGF-CC and PDGF-DD, and which engage with the receptor tyrosine kinases, PDGF receptor (PDGFR)-α and PDGFR-β (124). Cancer cells frequently express PDGFs which can trigger autocrine and paracrine PDGFR signaling to promote tumor growth, proliferation, stromal recruitment, angiogenesis, epithelial to mesenchymal cell transition, and metastasis (125).

PDGF-DD was identified as a ligand for NKp44 by screening a library of secreted proteins with NKp44 GFP reporter cells (67). PDGF-DD induced NK cell degranulation and the secretion of IFN-γ and TNF-α from NK cells and ILC1 as well as TNF-α secretion from ILC3 in agreement with a previous study (67, 126). IFN-γ and TNF-α secreted by NK cells stimulated with PDGF-DD could induce cell cycle arrest in melanoma, ovarian, and breast tumor cell-lines. Thus, innate immune cells have the capacity to sense the expression of GFs by tumor or tumor-associated stroma cells and potentially even infected or developing tissues to evoke NK cell responses. Remarkably, the upregulation of PDGF-DD-induced NK cell cytokines and chemokines and the downregulation of tumor cell cycle genes correlated with *NCR2* expression and was associated with greater survival in a cohort of glioblastoma patients. Moreover, transgenic expression of NKp44 in mouse NK cells resulted in greater control of tumors expressing PDGF-DD showing that whilst PDGF-DD can support tumor growth it also exposes tumor cells to NK cell immunosurveillance via NKp44. The ability of NK cells and ILCs to engage in GF surveillance via NKp44 is a new immunological paradigm that remains to be fully explored (67). Interestingly, a polymorphism in *PDGFD* is associated with serum IFN-γ levels in humans (127), suggesting the PDGF-DD/NKp44 interaction may play wider biological roles beyond cancer (Table 2).

**Table 2:** Disease and biological trait association of the NCRs and their ligands.

**NCR gene**	**Gene location**	**Disease association/reported trait**	**Reference (PubMed ID)**	**NCR Ligand gene**	**Gene location**	**NCR ligand disease association**	**Reference (PubMed ID)**
*NCR1*	19q13.42	Blood protein levels	29875488 30072576	*KMT2E* (parental gene for NKp44L)	7q22.3	Intelligence (MTAG)	29326435
						Educational attainment (MTAG)	30038396
						Educational attainment (years of education)	30038396
						Cognitive performance (MTAG)	30038396
				*PCNA*	20p12.3	No data available	
				*VMAC* (vimentin)	19p13.3	Post bronchodilator FEV1/FVC ratio	26634245
				*CFP*	Xp11.23	No data available	
*NCR2*	6p21.1	Alzheimer's disease biomarkers (p-tau measurement)	23562540	*PDGFD*	11q22.3	Interferon gamma levels	27989323
						Corticosteroid-induced adrenal suppression (peak cortisol < 350 nmol/L)	29551627
		Total ventricular volume (brain measurement)	21116278			Corticosteroid-induced adrenal suppression (peak cortisol < 500 nmol/L)	29551627
						LDL cholesterol	21347282
		Telomere length	29151059			Blood protein levels	29875488 30072576
						3-hydroxypropylmercapturic acid levels in smokers	26053186
				*SDC4* (Syndecan-4)	20q13.12	Bacterial meningitis	28928442
						Psoriasis	20953189
						Velopharyngeal dysfunction	29855589
						Lung function (FEV1/FVC)	30595370
				*NID1*	1q42.3	Urate levels in overweight individuals	25811787
						Cutaneous nevi	21478494
						Blood protein levels	30072576
						Waist-hip ratio	30595370
						Facial morphology (factor 20)	28441456
*NCR3*	6p21.33	C3 and C4 complement levels	23028341	*BAG6*	6p21.33	Small cell lung carcinoma	28604730
						Diastolic blood pressure	28739976 21909115
		Susceptibility to shingles	28928442			Systolic blood pressure	28739976 27618447
		Blood protein levels	29875488			Feeling worry	29500382
		Mosquito bite reaction, itch intensity measuremet	28199695			Hypertension	21909115
						Neuroticism measurement	29500382
						Psoriatic arthritis	30552173
		Neutrophil collagenase measurement	28240269			Urinary tract infection frequency	28928442
		Allergic disease (asthma, hay fever or eczema)	29083406	*LGALS3* (Galactin-3)	14q22.3	Blood protein levels	30072576
						Protein biomarker	23056639
						No data available	
		Menopause (age at onset)	29773799	*NCR3LG1* (*B7-H6*)	11p15.1		
		Laryngeal squamous cell carcinoma	25194280				
		Crohn's disease	22936669				
		Pulmonary function	23284291 28166213 21946350				
		IgG glycosylation	23382691				
		Height	23563607				
		Type 1 diabetes and autoimmune thyroid diseases	25936594				
		Weight	19079260				
		Diastolic blood pressure	28135244				
		Cold sores	28928442				
		Pneumonia	28928442				
		Strep throat	28928442				
		Tonsillectomy	28928442				
		Educational attainment (MTAG)	30038396				
		Self-reported mathematical ability	30038396				
		Highest math class taken (MTAG)	30038396				
		Heel bone mineral density	30598549				

### Nidogen-1

The ECM protein, Nidogen-1 (NID1, also known as Entactin) was identified as an NKp44 ligand, in another screen to identify soluble ligands that might regulate NCR activity and NK cell function (68). NID1 is an essential component of the basement membrane (BM) where it plays a role in BM assembly and stabilization, as well as adhesion between cells and the ECM (128). Soluble NID1 can be detected in the serum from patients with ovarian and lung cancer that may be derived from proteolytic degradation by cathepsin-S (CatS) (129, 130). Thus, it is conceivable that the release of NID1 into extracellular fluids may regulate the activity of activated NK cells in the blood or at specific tissue sites. Indeed, CatS-degradation and release of NID1 was proposed to reflect the loss of BM integrity, which is frequently associated with invasive tumors. Soluble NID1 was able to inhibit cytokine secretion mediated by PDGF-DD or cross-linking with anti-NKp44 mAbs. Consequently, the release of soluble NID1 was proposed to be a novel immunosuppressive mechanism exploited by tumor cells to evade NK cell surveillance (68).

### Galectin-3

Galectin-3 is a β-galactoside-binding lectin that has been reported to be a critical immune regulator in the tumor microenvironment (131). Galectin-3 can be expressed in the cytoplasm, nucleus, cell-surface, or extracellularly depending on the cell type and proliferative status. For example, extracellular galectin-3 can facilitate metastasis by promoting cell adhesion, invasiveness and immune evasion.

The genetic manipulation of galectin-3 expression levels in tumor cells showed that downregulation of galectin-3 lead to tumor growth inhibition, whereas the upregulation of galectin-3 led to enhanced tumor growth in human cervical and breast cancer models. Moreover, soluble galectin-3 released from tumor cells was found to bind specifically to NKp30 thereby inhibiting NKp30-mediated cytotoxicity. Thus, it was proposed that the secretion of galectin-3 by tumors represents a novel pathway to escape NKp30-mediated NK cell immunosurveillance (85).

## NCR-Mediated Control of Tumoricidal Pathways

The NCRs have been proposed to bind to many cellular ligands which are implicated in NK cell surveillance of tumor cells. Many of these interactions have been shown to evoke the cytotoxic and cytokine-secreting functions of NK cells. However, it is also possible that the NCRs may regulate other anti-tumor pathways. For melanoma metastases, NK cells were attributed to play a major role (99). However, ILC1 are phenotypically closely related to conventional NK cells as both these cell-types express T-bet and secrete IFN-γ (132, 133). ILC1 are characterized by higher TRAIL expression than NK cells due to tissue-imprinting by TGF-β (134). TRAIL (TNFSF10) can bind to TRAIL receptors (TRAIL-Rs), such as TRAIL-R1 in humans and TRAIL-R2 in humans and mice, which carry death domains that can induce caspase-8-mediated apoptosis of TRAIL-R^+^ tumor cells. TRAIL expression can be induced on NK cells activated by various cytokines and interferons (135–137) and several reports have described NK cell-mediated clearance of TRAIL-sensitive transplantable tumors (135, 138), chemical-induced fibrosarcoma (139), liver metastases (135, 139, 140), and hematological malignancies (141).

NKp46 was shown to regulate TRAIL surface expression resulting in the diminished cytotoxicity of TRAIL-R^+^ target cells by NKp46-deficient NK cells and ILC1 (142, 143). The NKp46-mediated pathway that regulates surface TRAIL expression remains to be fully characterized but appears to involve a post-transcriptional mechanism (142, 143). Whether NKp46 can regulate surface TRAIL expression when expressed by other immune cell subsets, such as γδ T cells and CD8^+^ T cells that have been expanded with IL-15, remains to be determined. These data show that NKp46 can endow NK cells or ILC1 with the ability to lyse TRAIL-sensitive tumors as well as contribute to TRAIL-mediated immunoregulation of infectious diseases, tissue inflammation (144–146), and potentially autoimmunity. It will also be interesting to see which NKp46 ligands can induce surface TRAIL expression in NK cells and ILC1 and the functional consequence for TRAIL-sensitive tumors *in vivo*.

### Control of Malignancy by Unconventional NCR^+^ Lymphocyte Subsets

Until recently NCR expression was considered to be restricted to NK cells. The NCRs have now been shown to be expressed by CD8^+^ T cell populations and γδ T cells expanded with IL-15, in addition to ILC1 and subsets of ILC3. Moreover, antibody-mediated stimulation of NKp44 and NKp46 on “NK-like” IL-15-expanded IEL CTL elicits the secretion of IFN-γ showing that these NCRs can function independently of TCR stimulation in T lymphocytes (31). Moreover, a population of NKp30^+^ CD8^+^ T cells with anti-tumor potential was shown to be induced by IL-15 (94). These NKp30^+^ CD8^+^ T cells exhibited high “NK-like” anti-tumor activity and NKp30 synergized with TCR signaling to control tumor growth in a preclinical xenograft mouse model.

Murine γδ T cells do not express NKp46, however, all three NCRs are expressed on human γδ T cells following continuous stimulation with TCR agonists or mitogens in the presence of IL-2 or IL-15 (30). Interestingly, NCR induction was mostly restricted to the Vδ1^+^ T cell subset and not Vδ2^+^ T cells. Like NK cells, the NCR^+^ Vδ1^+^ T cells were highly cytolytic against primary leukemia cells and tumor cell-lines in redirected cytotoxicity assays and NCR triggering also enhanced the expression of IFN-γ. Based on these observations, IL-15 expanded populations of “NK-like” CD8^+^ or Vδ1^+^ T cells that express NCRs have tremendous potential to be used clinically for adoptive cancer immunotherapy (147). The acquisition of NCRs by Vδ1^+^ T cells and CD8^+^ T cells is thought to require strong TCR activation, which is consistent with the oligoclonal expansion of gut IEL CTL in celiac disease that express a highly restricted TCR repertoire (31).

Although unconventional NCR^+^ lymphocyte subsets have been implicated in tumor surveillance they may also promote immunosuppression and facilitate tumor immune evasion. For example, a unique CD3^−^CD56^+^ ILC population was recently described that inhibits tumor-infiltrating lymphocytes (TILs) from high-grade serous ovarian carcinomas (148). The CD3^−^CD56^+^ ILC population was associated with a reduced TIL expansion and altered TIL cytokine production. This novel population of CD3^−^CD56^+^ ILCs exhibited low cytotoxic potential, secreted IL-22, and exhibited a transcriptional profile overlapping that of NK cells and other ILCs. NKp46 was highly expressed by the regulatory CD3^−^CD56^+^ ILC population and NKp46 signaling promoted the ability to suppress TIL expansion in co-culture (148).

Group 2 ILCs (ILC2s) secrete large amounts of type 2 cytokines, such as IL-5, IL-9, and IL-13, which promote alternative macrophage activation, eosinophilia, and goblet cell hyperplasia to limit parasite infection (149, 150). Increased levels of hyperactivated ILC2s expressing NKp30 and Chemoattractant receptor-homologous molecule expressed on Th2 cells (CRTH2) are found in patients with peripheral acute promyelocytic leukemia (APL). APL blasts were found to express high levels of surface B7-H6 and prostaglandin D2 (PGD2) that bind to NKp30 and CRTH2 (151) on ILC2s, respectively, to drive potent IL-13 secretion and activation of IL-13R^+^ myeloid-derived suppressor cells (152). Disruption of this tumor immunosuppressive axis by specifically blocking PGD2, IL-13, and NKp30 signaling partially normalized ILC2 and MDSC levels resulting in enhanced survival in leukemic mice (152).

## NCRs and their Ligands in Infectious Disease

The central role of the NCRs in resistance to infectious disease is exemplified by the susceptibility of NKp46-deficient mice to infection with different microorganisms. For example, human metapneumovirus (HMPV) causes acute respiratory tract infections in infants and children worldwide that can be fatal in immunosuppressed hosts. HMPV-infected cells express an unidentified ligand for NKp46 and NKp46-deficient mice are more susceptible to HMPV infection (153). The NCRs have also been shown to contribute to humoral immune responses and the generation of protective pathogen-specific antibodies. For example, deficiency in NKp46 resulted in the impaired maturation, function, and migration of NK cells to regional lymph nodes of mice infected with murine cytomegalovirus (MCMV). This was accompanied by a reduction in CD4^+^ T cell activation and follicular helper T cell generation leading to diminished B cell maturation in germinal centers and reduced titers of MCMV-specific antibodies (154).

Given the central role of NCRs in resistance to infectious disease, it is unsurprising that pathogens have evolved mechanisms to avoid NCR recognition. Herpesviruses are DNA viruses that have evolved various strategies of immune evasion as exemplified by their ability to establish latent infection with limited transcription of viral genes. Cells infected with human cytomegalovirus (HCMV) and human herpesvirus 6B (HHV6B) can express the NKp30 ligand, B7-H6. Both HMCV and HHV6B have evolved strategies to downregulate B7-H6 and ligands for other activating NK cell receptors, such as NKG2D, from the cell-surface, thus impairing NK cell recognition of virus-infected cells (155, 156). In the case of HCMV infection, HCMV expressed two gene products US18 and US20, that interfere with B7-H6 surface expression by promoting the lysosomal degradation of B7-H6 (155).

Many of the genes for the NCRs and their ligands are associated with infectious diseases highlighting the importance of the NCRs for pathogen recognition (Table 2). For example, *NCR2* is associated with chronic periodontitis in pregnant women and *NCR3* is associated with susceptibility to cold sores and shingles (Table 2). Here we document various NCR ligands that have been reported to play a role in pathogen surveillance by NK cells and other NCR^+^ immune cell subsets (Figure 2).

### Hemagglutinins and Hemagglutinin Neuraminidases

Even though the NCRs were first identified based on the ability to lyse certain tumor cell-lines, some of the first NCR ligands identified were viral proteins. Hemagglutinins (HA) from influenza (53, 66) and hemagglutinin-neuraminidases (HN) from parainfluenza, Sendai, and Newcastle disease viruses (53, 55) expressed on infected cells were shown to bind to NKp46 and NKp44. HAs from poxviruses, such as vaccinia and ectromelia, were also identified as ligands for NKp30 and NKp46 (54). However, the NCRs do not bind to HA from measles virus, showing that whilst the NCRs are capable of recognizing a broad range of viral HA and HNs there is some selectivity, which is predominantly dependent on α-2,3- and α-2,6-sialylated *O*-glycans on NCRs (53, 55, 56).

The interaction of viral-encoded HA and HNs on the surface of infected cells results in NK cell activation via NKp46 and NKp44 (53, 55, 56) and binding of the HN of Newcastle disease virus to NKp46 results in activation of downstream signaling molecules in the ITAM pathway e.g., Syk and surface upregulation of TRAIL on murine NK cells (157). In support of these observations, mice deficient in NKp46 expression are more susceptible to infection with influenza A virus (158, 159). In contrast, the interaction of NKp30 with HA expressed on the cell-surface or shed from vaccinia-infected cells counteracted activation by NKp46 resulting in the inhibition of NK cell lysis (54), suggesting a novel pathway of viral immune escape via NKp30. Moreover, exposure of NK cells to influenza virions or soluble HA can result in lysosomal degradation of the CD3ζ signaling chain and a reduction of NK cell cytotoxicity mediated by NKp46 and NKp30 (160). In contrast, the cleavage of sialic acid residues from NKp46 by the influenza virus neuraminidase (NA) was proposed as a mechanism of immune evasion by disrupting NKp46-binding to the viral HA and inhibitors specific for the influenza NA enhanced NKp46 recognition and virus clearance (161).

Human parainfluenza virus type 3 (HPIV3) is a virus that causes various respiratory illnesses, such as pneumonia, croup, and bronchiolitis, during infancy and childhood. Even though reinfection with HPIV3 throughout life is common there is no development of immunological memory and currently no effective vaccine or anti-viral therapies available. Poor T cell proliferation is observed following HPIV3 infection, which may be responsible for the lack of memory associated with the virus. NK cells have been shown to induce T cell cycle arrest in a contact-dependent manner, which was shown to be dependent on NKp44 and NKp46 but not NKp30 recognition of the HPIV3 HN on HPIV3-infected monocytes co-cultured with allogeneic-mixed lymphocytes (57). Consequently, the authors proposed that the success of future vaccines to HPIV3 may require the generation of modified HN proteins that retain immunogenicity but do not interact with NKp44 or NKp46.

### Human Cytomegalovirus Tegument Protein pp65

Intracellular staining of human cytomegalovirus (HCMV) infected fibroblasts revealed an unidentified protein that interacted with NKp30-Fc fusion protein. Purified virions from HCMV-infected fibroblasts also bound to NKp30-Fc but not to control Fc-fusion proteins. These data suggested the unidentified viral protein that interacted with NKp30 may be incorporated into HCMV virus particles. The NKp30 Fc fusion was used to immunoprecipitate proteins from HCMV-infected fibroblasts and the HCMV-encoded tegument protein pp65 was subsequently identified as a ligand for NKp30 using mass spectrometry (79). HCMV pp65 was shown to bind directly to NKp30 and induce the dissociation of the signaling adaptor CD3ζ. HCMV-infected fibroblasts were found to be less susceptible to NK cell cytotoxicity compared to fibroblasts infected with a pp65-deficient HCMV or in the presence of blocking antibodies to NKp30. Thus, pp65 furnishes HCMV with a novel immune escape pathway by disrupting the surveillance of virus-infected cells by NKp30.

### Flavivirus Proteins

NKp44 Fc-fusion proteins have also been shown to interact with purified envelope protein from the flaviviruses, dengue virus (DV), and West Nile Virus (WNV) and WNV virus-like particles. NKp44 was shown to specifically bind to domain III of the envelope protein from WNV, which also bound to NK cells expressing high levels of NKp44 and the binding of infectious WNV and WNV-infected cells stimulated NK cell degranulation and IFN-γ secretion in an NKp44-dependent manner (74). Unlike the interaction with viral hemagglutinins, NKp44 binding to domain III of the DV and WNV envelope proteins was independent of glycan-linked sialic acid residues on NKp44.

### Bacterial NCR Ligands

In contrast to viral ligands, the NCRs have also been shown to bind directly to molecules encoded by bacteria and parasites in addition to host molecules exposed on the surface of cells infected with bacteria. For example, an Fc-fusion protein of NKp44 (NKp44-Fc), but not NKp30 or NKp46, was shown to bind to *Mycobacterium bovis* (*M. bovis*), *M. tuberculosis, Nocardia farcinica*, and *Pseudomonas aeruginosa* (75, 76). NKp44 was specifically shown to bind to cell wall components, such as arabinogalactan-peptidoglycan (mAGP) as well as to mycolic acids and arabinogalactan and derivatives, from *M. tuberculosis*. Whilst a direct role in NK cell cytotoxicity could not be demonstrated, anti-NKp44 antibodies inhibited mAGP and BCG-induced CD69 expression on IL-2-activated NK cells, suggesting NKp44 recognition of bacterial wall components may sustain NK cell activation during infection with *M. tuberculosis* (76). In contrast to NKp44, NKp46 was reported to play a dominant role in the lysis of mononuclear phagocytes infected with *M. tuberculosis* (59). NKp46 was subsequently shown to recognize vimentin expressed on the surface of cells infected with *M. tuberculosis* (60). Antibodies to vimentin inhibited NK cell lysis of monocytes infected with *M. tuberculosis*, whereas transfection of vimentin enhanced NK cell lysis of Chinese hamster ovary cells (60).

*Fusobacterium nucleatum* and *Porphyromonus gingivalis* are involved in the pathogenesis of periodontitis. The severity of periodontal disease is determined by the host's immune response. Alveolar bone loss occurred in wild-type mice but was minimal in NKp46-deficient mice in an oral infection model using *Fusobacterium nucleatum* or *Porphyromonus gingivalis* (61). Expression of an NKp46 ligand by *F. nucleatum* was demonstrated by the binding of a NKp46-Fc and the activation of NKp46 reporter cells as well as the secretion of TNF-α from wild-type but not NKp46-deficient NK cells (61). Interestingly, *NCR2*, but not *NCR1*, is associated with chronic periodontitis in humans (Table 2) (162), which could reflect cross-talk in NCR signaling (163).

### Parasite NCR Ligands

Parasite infection has also been shown to induce the expression of NCR ligands. Fc fusion proteins of NKp30 and NKp46 were shown to specifically bind to the Duffy binding-like (DBL)-1 α domain of *Plasmodium falciparum* erythrocyte membrane protein-1 (PfEMP-1), which is expressed on the cell-surface of parasitized erythrocytes (58). NK cells lysed erythrocytes infected with *P. falciparum*, which was blocked by antibodies to NKp30 and NKp46 with the greatest inhibition observed when both antibodies were used in combination.

### Fungal NCR Ligands

NKp46 and NKp30 have been shown to interact with fungal ligands. Both mouse and human NKp46 were shown to bind to the emerging fungal pathogen, *Candida glabrata* but not to *C. albicans, C. parapsilosis, C. krusei*, or *Cryptococcus gattii* (63). Fc fusion proteins from human and mouse NKp46 were specifically shown to bind to the *C. glabrata* adhesins Epa1, Epa6, and Epa7. The Epa proteins are lectins that bind to glycans to facilitate the *C. glabrata* infection of mammalian cells and mutation of *O*-linked glycosylation of Threonine 225 of mouse and human NKp46 abolished binding to *C. glabrata* Epa proteins (63). Moreover, the clearance of systemic *C. glabrata* infection was impaired in NKp46-deficient mice.

NKp30 mediates the recognition of *Cryptococcus neoformans* and *C. albicans* resulting in the formation NK cell-fungal conjugates and direct fungal killing (164). NKp30 was specifically shown to bind to the fungal cell wall component β-1,3 glucan to stimulate the polarization of cytotoxic granules in NK cell-fungal conjugates, which enhanced the release of perforin required for fungal cytotoxicity (86). Rather than blocking the interaction between NK cells and fungi, soluble β-1,3 glucan enhanced fungal killing (86). Fungal infections are the leading cause of death in AIDS patients and NK cells from HIV-infected patients had reduced expression of NKp30. IL-12 and β-1,3 glucan restored NK cell expression of NKp30 and fungal killing by NK cells (86, 164). The activation of src family kinases by NKp30 synergized with β1 integrin signaling and the activation of integrin-linked kinase (ILK) and Rac1, which converged into a central PI3 kinase signaling pathway that was required for fungal killing by NK cells (87).

### Complement Factor P (properdin)

The complement system is an evolutionary ancient component of the innate immune response. Complement factor P (CFP, also known as properdin) is a plasma glycoprotein that binds to microbial surfaces and is the only known positive regulator of the alternative pathway of complement (165). CFP stabilizes the C3- and C5-convertase enzyme complexes that ultimately leads to the formation of the membrane attack complex and target cell lysis. NKp46 was shown to bind to CFP. Patients deficient in CFP are more susceptible to lethal *Neisseria meningitidis* due to an inability to activate the alternative pathway of complement (166).

CFP was shown to bind to a recombinant NKp46 Fc-fusion protein and to activate NKp46 reporter cells but could not induce classical NK cell activation characterized by degranulation and IFN-γ secretion (64). Instead CFP induced the upregulation of genes encoding the leucine zipper protein FosB and the chemokine XCL1 (also known as lymphotactin). Thus, CFP binding to NKp46 was proposed to activate a non-canonical pathway of gene expression in NK cells. In agreement with patients deficient in CFP, NKp46 and ILC1 were required for mice to survive infection with *N. meningitidis*. Moreover, the beneficial effects of CFP supplementation for *N. meningitidis* infection were dependent on NKp46 and ILC1 (64).

### Unconventional Roles for NCRs in the Control of Infection

NKp30 is also expressed on γδ T cells (30) bearing TCR Vδ1 but not TCR Vδ2. Circulating γδ T cells are normally devoid of NCR expression. However, all three NCRs were induced on γδ T cells following TCR engagement in conjunction with cytokines, such as IL-2 and IL-15 (30). Moreover, mAb cross-linking of NKp30 on NCR^+^ Vδ1 T cells, and to a lesser extend NKp44 and NKp46, induced the chemokines CCL1, CCL2 and CCL3, which suppressed HIV infection by binding the HIV co-receptor, CCR5 (167).

## NCRs and their Ligands in Development

In steady state, human uterine NK (uNK) cells maintain endometrial arteries (168). However, during pregnancy, uterine NK cells maintain key developmental processes, such as trophoblast invasion, remodeling uterine vasculature, and the promotion of fetal growth (169). The tissue origins of uNK remain obscure and are thought to constitute a heterogenous population derived from local endometrial NK cells as well as conventional NK cells recruited from the circulation (169, 170). Uterine NK cells possess limited cytotoxicity and actively secrete cytokines, chemokines, GFs, and angiogenic factors required for the correct remodeling of maternal spiral arteries and likely function by promoting the recruitment of invading trophoblast cells and promoting angiogenesis during pregnancy (171).

The NCRs have been reported to be expressed by uNK (171–173) and the ligands for NKp30 and NKp44 are also expressed by trophoblast cells (171). Consequently, trophoblast cells have the potential to directly stimulate uNK functions via the NCRs and ligation of the NCRs with specific antibodies resulted in the secretion of cytokines, chemokines, GFs, and angiogenic factors by uNK (171–173). Thus, NCR expression endows uNK with the capacity to orchestrate key developmental processes at the fetal-maternal interface following interactions with trophoblast cells or other NCR ligands expressed in decidua. Uterine NK and peripheral blood NK (pNK) operate in different tissue microenvironments and also differ in NCR expression. For example, resting pNK do not express NKp44 unlike uNK. The decidual stroma expresses the cytokines IL-15, IL-18, and TGF-β, that are required for successful pregnancy. Uterine NK were found to express distinct NKp30 and NKp44 splice variants compared to pNK, characterized by predominant expression of the inhibitory NKp30c and NKp44-1 isoforms and lower amounts of the activating NKp30a/b and NKp44-2/3 isoforms (174). A combination of IL-15, IL-18, and TGF-β shifted the pNK NCR splice variant expression profile toward that of uNK suggesting the decidual tissue microenvironment can confer a decidual-like uNK phenotype on pNK (174).

## NCRs and their Ligands in Allergy

Allergic disease is caused by inappropriate immune responses to harmless antigens driven by type 2 cytokines. Very few papers have documented a role for NK cells or the NCRs in allergic disease. However, recent studies have suggested that NK cells and the NCRs may be important in allergy due to the immunoregulatory activity of NK cells on other immune cell subsets (175) and NK cells have also been shown to drive allergic responses independently of T and B cells (176, 177). NKp46-deficient mice were shown to have reduced skin and airway hypersensitivity (178). In a delayed type IV hypersensitivity model of airway inflammation, the number of IFN-γ producing NK cells and the proliferation of OVA-specific T cells was reduced following intranasal OVA challenge in NKp46-deficient mice, suggesting that differences in the stimulation of T cells resulting in an altered cytokine profile upon challenge with ovalbumin (OVA) (178). These results suggested that NKp46 signaling enhances allergic responses. In contrast, a separate study using a delayed type I hypersensitivity model, showed that NKp46 signaling suppressed the development of airway eosinophilia following intranasal OVA challenge (179). It will be interesting to see which NKp46-expressing cell types, such as NK cells, ILC1 or ILC3, might be responsible for either suppressing (179) or augmenting (178) airway inflammation in models of type I and type IV hypersensitivity, respectively.

NCR expression by other lymphocyte subsets may also regulate allergic response. For example, whilst ILC2s secrete type 2 cytokines to limit parasite infection they are also important contributors to allergic inflammation (149, 150). Activated human ILC2s can express NKp30 and stimulation of ILC2s with B7-H6 induced the rapid production of type 2 cytokines, such as IL-13, which was blocked by anti-NKp30 blocking mAb and galectin-3 (180). Higher expression of B7-H6 is observed in skin lesion biopsies from patients with atopic dermatitis and stimulation of keratinocytes with proinflammatory as well as type 2 cytokines upregulated B7-H6 expression leading to enhanced ILC2 production of type 2 cytokines. Thus, the NKp30-B7-H6 interaction is a novel cell contact-dependent interaction that can mediate ILC2 activation and is a potential target for the development of novel therapeutics for atopic dermatitis and possibly other atopic diseases (180).

## NCRs and Their Ligands in Autoimmunity

NK cell have also been implicated in the development of autoimmune diseases, such as type 1 diabetes (181–184). NKp46-deficient mice were reported to be resistant to the development diabetes induced by streptozotocin and a soluble NKp46-Fc protein blocked the development of diabetes in mice (62). NKp46 was suggested to play a dominant role in NK cell cytotoxicity of human pancreatic β-cells through the recognition of an as yet unidentified NKp46 ligand expressed in the β-cell insulin granules (97). Anti-NKp46 mAbs that can induce the downregulation and lysosomal degradation of mouse and human NKp46 have now been developed, which may be effective in the treatment of diabetes (185, 186).

Primary Sjogren's syndrome (pSS) is a chronic autoimmune disease characterized by lymphocytic exocrinopathy that can affect up to 0.1 to 0.6% of the general population. An excess accumulation of NK cells in minor salivary glands of pSS patients is correlated with the severity of exocrinopathy. B7-H6, the ligand for NKp30, is expressed in minor salivary glands and a higher cell-surface expression of NKp30 on circulating NK cells is observed in pSS patients compared to healthy controls (187). A case control study of genetic polymorphisms revealed that a single nucleotide polymorphism (SNP) rs11575837 (*G* > *A*) in the promoter region of *NCR3* was associated with reduced transcription and protection from pSS. These findings suggested that NK cells may promote NKp30-dependent inflammation in salivary glands and blockade of the NKp30-B7-H6 interaction may represent a novel clinical target for pSS (187).

## NCR-Based Cancer Immunotherapies

In addition to the development of therapeutic antibodies to the NCRs for potential use in the treatment of autoimmune diseases, such as diabetes (see above), several reports are now beginning to provide evidence that the NCRs and their ligands can be successfully targeted for cancer immunotherapy. Moreover, these systems have shown to be effective in various pre-clinical mouse tumor models highlighting the tremendous potential of NCR-based strategies for tumor immunotherapy.

### Virotherapy

Reovirus is a double-stranded RNA virus that infects much of the population during childhood causing mild or subclinical infections. Interestingly, reovirus efficiently infects tumor cells and is currently be tested in human clinical trials as a potential cancer virotherapy although the mechanism whereby reovirus infected tumor cells are recognized and eliminated by the immune system is not well-understood. The reovirus σ1 protein forms an elongate trimer that serves as a viral attachment protein (188). Human and mouse NKp46 were shown to bind to reovirus σ1 protein in a sialic acid-dependent manner that led to NK cell activation *in vitro* (189) (Figure 2). NK cells and NKp46 were shown to be essential for the clearance of reovirus infection from the lungs of infected mice and for the success of reovirus-based tumor therapy (189).

### Bi-Specific T Cell Engagers (BiTE)

The NKp30/B7-H6 interaction has been targeted using various therapeutic strategies. One approach has been to target T cells to B7-H6 expressing tumors using a BiTE that incorporated a single-chain F_V_ (ScFV) to B7-H6 fused to an anti-CD3ε ScFV. The B7-H6-specific BiTE promoted T cell cytotoxicity and IFN-γ secretion against B7-H6^+^ tumor cells, which enhanced the survival of lymphoma-bearing mice and decreased the tumor burden of melanoma- and ovarian cancer-bearing mice (190). Similar conceptual approaches, have involved fusing the B7-H6 ectodomain (with the aim of engaging NKp30 on NK cells) to either an anti-Human Epidermal Growth factor 2 (HER2) scFv to enhance NK cell killing of HER2^+^ breast cancers (191) or an anti-CD20 scFv to target lymphomas (192, 193). Such reagents were reported to be effective at nM concentrations and to enhance ADCC mediated by therapeutic antibodies, such as trastuzumab, cetuximab, and rituximab (191–193).

### NCR-Based Chimeric Antigen Receptors (CARs)

Yet another therapeutic strategy has been to engineer chimeric antigen receptor (CAR) constructs that incorporate either the NKp30 ectodomain (NKp30-CAR) or an anti-B7-H6 scFv (B7H6-specific CAR) with TM domains fused to the CD3ζ or and/or CD28 cytoplasmic signaling domains for cell-surface expression in T or NK cells intended for use in adoptive cancer immunotherapy (194–196). NKp30-CAR and B7-H6-specific CAR T cells elicited robust cellular cytotoxicity and IFN-γ secretion when co-cultured with B7-H6^+^ tumor cells. Dendritic cells (DCs) also enhanced IFN-γ secretion from NKp30-CAR expressing T cells, whereas B7-H6-specific CAR T cells exhibited little self-reactivity to DCs and monocytes expressing B7-H6 ligands. The adoptive transfer of T cells expressing NKp30-CAR or B7-H6-specific CAR T cells enhanced the survival of mice bearing RMA lymphoma cells transduced with B7-H6. Moreover, mice that remained tumor-free, were resistant to subsequent rechallenge with B7-H6-deficient parental RMA tumor cells (194, 195). NKp44- and NKp46-derived CARs have also been developed and were shown to elicit clinical efficacy for various solid tumors that express ligands for these receptors (197, 198).

## Discussion

The NCRs were originally identified as activating receptors expressed on NK cells. However, it is now apparent the NCRs are expressed by ILCs in addition to adaptive Vδ1^+^ and CD8^+^ T cells. The NCRs have been shown to synergize with TCR signaling as well as function independently on T cells. Thus, like NK cells, Vδ1^+^ and CD8^+^ T cells expressing NCRs hold potential for adoptive cancer immunotherapy (147, 199). It will be interesting to see how NCR^+^ Vδ1 T cells and CD8^+^ T cell functions are regulated by the range of NCR ligands now reported in the literature.

The NCRs can interact with soluble as well as membrane bound ligands. Such polyfunctionality is not uncommon in the immune system (25). For example, the activating immunoreceptor OSCAR signals via FcRγ and possesses two Ig-like domains similar to NKp46 and genes for both proteins are encoded in the Leukocyte Receptor Complex (LRC), suggesting a common evolutionary origin (200). OSCAR interacts with at least three different types of ECM collagens to costimulate osteoclastogenesis (201–203) and also binds to the secreted collagen-like lectin, Surfactant Protein D (SP-D) (204). The LRC-encoded immunoreceptors, LAIR-1 and LAIR-2, also bind to multiple ECM collagens as well as SP-D (205–207). Moreover, NKG2D, a member of the C-type lectin family of activating NK cell receptors, is highly promiscuous and binds to a number of cell-surface and shed MHC class I-like ligands to regulate NK cell functions (199, 208–211). However, in contrast to OSCAR, LAIR-1 and−2, and NKG2D, which all bind structurally similar surface or soluble ligands, respectively, the NCRs have been reported to bind a structurally diverse set of ligands. Thus, many open questions regarding NCR ligand binding still remain, such as how can a given NCR bind to such a broad range of structurally diverse ligands? Conversely, how can the structurally diverse NCRs all bind the same classes of ligands, such as viral HAs and cellular HS GAGs? Structural studies are clearly required to resolve many of these outstanding questions and may reveal fascinating insights into the polyfunctionality of the NCRs as well as intriguing comparisons for other immunoreceptor families.

Such a structurally diverse set of soluble and surface bound NCR ligands might also be expected to induce different strengths and duration of activating or inhibitory NCR signaling that may regulate NK cell activity in different tissues and inflammatory settings. Recent data have raised the importance of secreted ligands in the “alternative activation” of NK cells and the NCRs are no exception (64, 67, 210). For example, PDGF-DD has been reported to bind to NKp44 to induce the secretion of IFN-γ and TNF-α and chemokines, such as CCL1, CCL3, CCL4, XCL1 and XCL2 (67), whereas CFP binding to NKp46 only elicits the expression of XCL1 (64). Firstly, these data challenge the view that stimulation with soluble or shed ligands invariably leads to the desensitization of activating receptor pathways and NK cell inhibition. Secondly, the range of different response to soluble ligands suggests the NCR interaction with each ligand may be tailored to the tissue microenvironment for a specific function or to avoid immunopathology. For example, CFP binding to NKp46, is critical for resistance to *N. meningitidis* infection, which causes septicaemia and meningitis (64). It is conceivable that the reduced NK cell activation induced by the CFP/NKp46 interaction may be desirable in the meninges given the proximity of the inflammatory response to the brain. Further research into the molecular basis and cell biology of NCR signaling for surface-bound and soluble ligands is warranted to fully understand how soluble ligands, such as PDGF-DD and CFP, as well as shed ligands, such as MULT1, (210), evoke appropriate NK cell activation in different organs and tissues and within the tumor microenvironment (212). Such approaches will inform methods to enhance NK cell targeting and to stimulate or inhibit their functions *in vivo* and will lead to the development of novel clinical interventions (212, 213). For example, CARs have already been engineered for soluble growth factors, such as TGF-β, that function in T cells and promote anti-tumor responses *in vivo* (67, 214, 215).

Many of the proposed NCR ligands are normally localized to the nucleus or cytoplasmic proteins, such as BAT3, NKp44L, and PCNA. However, the cellular pathways responsible for the cell-surface localization of these ligands and their selective upregulation in malignant vs. non-malignant cells still remain to be characterized in molecular detail. These questions are critical for understanding how the NCR ligands regulate the activity of NK cells and other NCR^+^ immune cell subsets in different tissues and pathological processes. Interestingly, the NCRs and their ligands are associated with a diverse range of diseases and biological traits (Table 2). It might be expected that many of these disease associations are independent of the NCRs and result from changes to the primary biological function of the NCR ligand in question. However, it is also feasible that the NCRs and their ligands could play a pathogenetic role for some of these aforementioned diseases. Further research into the role of the NCRs and their ligands that are genetically associated with disease should be encouraged and will provide novel insights into the role of tissue NK cells for diseases that may have an as yet unrecognized immunopathological component.

## Author Contributions

AB, CM, and MC authored and edited the manuscript. CM contributed figures and tables.

### Conflict of Interest Statement

The authors declare that the research was conducted in the absence of any commercial or financial relationships that could be construed as a potential conflict of interest.

## References

[1] Aw Yeang HX Piersma SJ Lin Y Yang L Malkova ON Miner C. Cutting edge: human CD49e- NK cells are tissue resident in the liver. J Immunol. (2017) 198:1417–22. 10.4049/jimmunol.1601818 28093522 PMC5296254

[2] Crinier A Milpied P Escalière B Piperoglou C Galluso J Balsamo A. High-dimensional single-cell analysis identifies organ-specific signatures and conserved NK cell subsets in humans and mice. Immunity. (2018) 49:971–86 e5. 10.1016/j.immuni.2018.09.009 30413361 PMC6269138

[3] Sojka DK Plougastel-Douglas B Yang L Pak-Wittel MA Artyomov MN Ivanova Y. Tissue-resident natural killer (NK) cells are cell lineages distinct from thymic and conventional splenic NK cells. eLife. (2014) 3:e01659. 10.7554/eLife.01659 24714492 PMC3975579

[4] Sharkey AM Xiong S Kennedy PR Gardner L Farrell LE Chazara O. Tissue-specific education of decidual NK cells. J Immunol. (2015) 195:3026–32. 10.4049/jimmunol.1501229 26320253 PMC4574523

[5] Kiessling R Klein E Wigzell H. “Natural” killer cells in the mouse. I. Cytotoxic cells with specificity for mouse Moloney leukemia cells. Specificity and distribution according to genotype. Eur J Immunol. (1975) 5:112–7. 10.1002/eji.1830050208 1234049

[6] Herberman RB Nunn ME Lavrin DH. Natural cytotoxic reactivity of mouse lymphoid cells against syngeneic acid allogeneic tumors. I. Distribution of reactivity and specificity. Int J Cancer. (1975) 16:216–29. 50294 10.1002/ijc.2910160204

[7] Ljunggren HG Kärre K In search of the “missing self”: MHC molecules and NK cell recognition. Immunol Today. (1990) 11:237–44. 2201309 10.1016/0167-5699(90)90097-s

[8] Bix M Liao NS Zijlstra M Loring J Jaenisch R Raulet D. Rejection of class I MHC-deficient haemopoietic cells by irradiated MHC-matched mice. Nature. (1991) 349:329–31. 10.1038/349329a0 1987491

[9] Liao NS Bix M Zijlstra M Jaenisch R Raulet D. MHC class I deficiency: susceptibility to natural killer (NK) cells and impaired NK activity. Science. (1991) 253:199–202. 1853205 10.1126/science.1853205

[10] Karlhofer FM Ribaudo RK Yokoyama WM. MHC class I alloantigen specificity of Ly-49+ IL-2-activated natural killer cells. Nature. (1992) 358:66–70. 10.1038/358066a0 1614533

[11] Colonna M Samaridis J. Cloning of immunoglobulin-superfamily members associated with HLA-C and HLA-B recognition by human natural killer cells. Science. (1995) 268:405–8. 7716543 10.1126/science.7716543

[12] Wagtmann N Biassoni R Cantoni C Verdiani S Malnati MS Vitale M. Molecular clones of the p58 NK cell receptor reveal immunoglobulin-related molecules with diversity in both the extra- and intracellular domains. Immunity. (1995) 2:439–49. 7749980 10.1016/1074-7613(95)90025-x

[13] Colonna M Brooks EG Falco M Ferrara GB Strominger JL. Generation of allospecific natural killer cells by stimulation across a polymorphism of HLA-C. Science. (1993) 260:1121–4. 8493555 10.1126/science.8493555

[14] Malnati MS Peruzzi M Parker KC Biddison WE Ciccone E Moretta A. Peptide specificity in the recognition of MHC class I by natural killer cell clones. Science. (1995) 267:1016–8. 7863326 10.1126/science.7863326

[15] Lanier LL Yu G Phillips JH. Co-association of CD3 zeta with a receptor (CD16) for IgG Fc on human natural killer cells. Nature. (1989) 342:803–5. 10.1038/342803a0 2532305

[16] Vivier E Ackerly M Rochet N Anderson P. Structure and function of the CD16:zeta:gamma complex expressed on human natural-killer cells. Int J Cancer Suppl J Int Cancer Suppl. (1992) 7:11–4. 1428397

[17] Vivier E da Silva AJ Ackerly M Levine H Rudd CE Anderson P. Association of a 70-kDa tyrosine phosphoprotein with the CD16: zeta: gamma complex expressed in human natural killer cells. Eur J Immunol. (1993) 23:1872–6. 10.1002/eji.1830230821 8344348

[18] Pende D Parolini S Pessino A Sivori S Augugliaro R Morelli L. Identification and molecular characterization of NKp30, a novel triggering receptor involved in natural cytotoxicity mediated by human natural killer cells. J Exp Med. (1999) 190:1505–16. 10562324 10.1084/jem.190.10.1505 PMC2195691

[19] Sivori S Vitale M Morelli L Sanseverino L Augugliaro R Bottino C. p46, a novel natural killer cell–specific surface molecule that mediates cell activation. J Exp Med. (1997) 186:1129–36. 9314561 10.1084/jem.186.7.1129 PMC2211712

[20] Vitale M Bottino C Sivori S Sanseverino L Castriconi R Marcenaro E. NKp44, a novel triggering surface molecule specifically expressed by activated natural killer cells, is involved in non-major histocompatibility complex-restricted tumor cell lysis. J Exp Med. (1998) 187:2065–72. 9625766 10.1084/jem.187.12.2065 PMC2212362

[21] Pessino A Sivori S Bottino C Malaspina A Morelli L Moretta L. Molecular cloning of NKp46: a novel member of the immunoglobulin superfamily involved in triggering of natural cytotoxicity. J Exp Med. (1998) 188:953–60. 9730896 10.1084/jem.188.5.953 PMC3207313

[22] Dietrich J Nakajima H Colonna M. Human inhibitory and activating Ig-like receptors which modulate the function of myeloid cells. Microbes Infect. (2000) 2:323–9. 10.1016/S1286-4579(00)00294-X 10758410

[23] Foster CE Colonna M Sun PD. Crystal structure of the human natural killer (NK) cell activating receptor NKp46 reveals structural relationship to other leukocyte receptor complex immunoreceptors. J Biol Chem. (2003) 278:46081–6. 10.1074/jbc.M308491200 12960161

[24] Ponassi M Cantoni C Biassoni R Conte R Spallarossa A Pesce A. Structure of the human NK cell triggering receptor NKp46 ectodomain. Biochem Biophys Res Commun. (2003) 309:317–23. 10.1016/j.bbrc.2003.08.007 12951052

[25] Trowsdale J Jones DC Barrow AD Traherne JA. Surveillance of cell and tissue perturbation by receptors in the LRC. Immunol Rev. (2015) 267:117–36. 10.1111/imr.12314 26284474

[26] Walzer T Bléry M Chaix J Fuseri N Chasson L Robbins SH. Identification, activation, and selective *in vivo* ablation of mouse NK cells via NKp46. Proc Natl Acad Sci USA. (2007) 104:3384–9. 10.1073/pnas.0609692104 17360655 PMC1805551

[27] Diefenbach A Colonna M Koyasu S. Development, differentiation, and diversity of innate lymphoid cells. Immunity. (2014) 41:354–65. 10.1016/j.immuni.2014.09.005 25238093 PMC4171710

[28] Verrier T Satoh-Takayama N Serafini N Marie S Di Santo JP Vosshenrich CAJ. Phenotypic and functional plasticity of murine intestinal NKp46+ Group 3 innate lymphoid cells. J Immunol. (2016) 196:4731–38. 10.4049/jimmunol.1502673 27183613

[29] Stewart CA Walzer T Robbins SH Malissen B Vivier E Prinz I. Germ-line and rearranged Tcrd transcription distinguish bona fide NK cells and NK-like gammadelta T cells. Eur J Immunol. (2007) 37:1442–52. 10.1002/eji.200737354 17492716

[30] Correia DV Fogli M Hudspeth K da Silva MG Mavilio D Silva-Santos B. Differentiation of human peripheral blood Vδ1+ T cells expressing the natural cytotoxicity receptor NKp30 for recognition of lymphoid leukemia cells. Blood. (2011) 118:992–1001. 10.1182/blood-2011-02-339135 21633088

[31] Meresse B Curran SA Ciszewski C Orbelyan G Setty M Bhagat G. Reprogramming of CTLs into natural killer-like cells in celiac disease. J Exp Med. (2006) 203:1343–55. 10.1084/jem.20060028 16682498 PMC2121214

[32] Ettersperger J Montcuquet N Malamut G Guegan N Lopez-Lastra S Gayraud S. Interleukin-15-dependent T-cell-like innate intraepithelial lymphocytes develop in the intestine and transform into lymphomas in celiac disease. Immunity. (2016) 45:610–25. 10.1016/j.immuni.2016.07.018 27612641

[33] Tang Q Grzywacz B Wang H Kataria N Cao Q Wagner JE. Umbilical cord blood T cells express multiple natural cytotoxicity receptors after IL-15 stimulation, but only NKp30 is functional. J Immunol. (2008) 181:4507–15. 18802053 10.4049/jimmunol.181.7.4507 PMC2614673

[34] Freud AG Zhao S Wei S Gitana GM Molina-Kirsch HF Atwater SK. Expression of the activating receptor, NKp46 (CD335), in human natural killer and T-cell neoplasia. Am J Clin Pathol. (2013) 140:853–66. 10.1309/AJCPWGG69MCZOWMM 24225754

[35] Bensussan A Remtoula N Sivori S Bagot M Moretta A Marie-Cardine A. Expression and function of the natural cytotoxicity receptor NKp46 on circulating malignant CD4+ T lymphocytes of Sézary syndrome patients. J Invest Dermatol. (2011) 131:969–76. 10.1038/jid.2010.404 21191411

[36] Yu J Mitsui T Wei M Mao H Butchar JP Shah MV. NKp46 identifies an NKT cell subset susceptible to leukemic transformation in mouse and human. J Clin Invest. (2011) 121:1456–70. 10.1172/JCI43242 21364281 PMC3069763

[37] Mattiola I Pesant M Tentorio PF Molgora M Marcenaro E Lugli E. Priming of human resting NK cells by autologous M1 macrophages via the engagement of IL-1β, IFN-β, and IL-15 pathways. J Immunol. (2015) 195:2818–28. 10.4049/jimmunol.1500325 26276870

[38] Cantoni C Bottino C Vitale M Pessino A Augugliaro R Malaspina A. NKp44, a triggering receptor involved in tumor cell lysis by activated human natural killer cells, is a novel member of the immunoglobulin superfamily. J Exp Med. (1999) 189:787–96. 10049942 10.1084/jem.189.5.787 PMC2192947

[39] Shemesh A Kugel A Steiner N Yezersky M Tirosh D Edri A. NKp44 and NKp30 splice variant profiles in decidua and tumor tissues: a comparative viewpoint. Oncotarget. (2016) 7:70912–23. 10.18632/oncotarget.12292 27765926 PMC5342598

[40] Lanier LL Corliss BC Wu J Leong C Phillips JH. Immunoreceptor DAP12 bearing a tyrosine-based activation motif is involved in activating NK cells. Nature. (1998) 391:703–7. 10.1038/35642 9490415

[41] Tomasello E Olcese L Vély F Geourgeon C Bléry M Moqrich A. Gene structure, expression pattern, and biological activity of mouse killer cell activating receptor-associated protein (KARAP)/DAP-12. J Biol Chem. (1998) 273:34115–9. 9852069 10.1074/jbc.273.51.34115

[42] Cantoni C Ponassi M Biassoni R Conte R Spallarossa A Moretta A. The three-dimensional structure of the human NK cell receptor NKp44, a triggering partner in natural cytotoxicity. Struct Lond Engl 1993. (2003) 11:725–34. 12791260 10.1016/s0969-2126(03)00095-9

[43] Allcock RJN Barrow AD Forbes S Beck S Trowsdale J. The human TREM gene cluster at 6p21.1 encodes both activating and inhibitory single IgV domain receptors and includes NKp44. Eur J Immunol. (2003) 33:567–77. 10.1002/immu.200310033 12645956

[44] De Maria A Ugolotti E Rutjens E Mazza S Radic L Faravelli A. NKp44 expression, phylogenesis and function in non-human primate NK cells. Int Immunol. (2009) 21:245–55. 10.1093/intimm/dxn144 19147838 PMC2645782

[45] Campbell KS Yusa S Kikuchi-Maki A Catina TL NKp44 triggers NK cell activation through DAP12 association that is not influenced by a putative cytoplasmic inhibitory sequence. J Immunol. (2004) 172:899–906. 14707061 10.4049/jimmunol.172.2.899

[46] Cortez VS Ulland TK Cervantes-Barragan L Bando JK Robinette ML Wang Q. SMAD4 impedes the conversion of NK cells into ILC1-like cells by curtailing non-canonical TGF-β signaling. Nat Immunol. (2017) 18:995–1003. 10.1038/ni.3809 28759002 PMC5712491

[47] Fuchs A Vermi W Lee JS Lonardi S Gilfillan S Newberry RD. Intraepithelial type 1 innate lymphoid cells are a unique subset of IL-12- and IL-15-responsive IFN-γ-producing cells. Immunity. (2013) 38:769–81. 10.1016/j.immuni.2013.02.010 23453631 PMC3634355

[48] Cella M Fuchs A Vermi W Facchetti F Otero K Lennerz JKM. A human natural killer cell subset provides an innate source of IL-22 for mucosal immunity. Nature. (2009) 457:722–5. 10.1038/nature07537 18978771 PMC3772687

[49] Cella M Otero K Colonna M. Expansion of human NK-22 cells with IL-7, IL-2, and IL-1beta reveals intrinsic functional plasticity. Proc Natl Acad Sci USA. (2010) 107:10961–6. 10.1073/pnas.1005641107 20534450 PMC2890739

[50] von Lilienfeld-Toal M Nattermann J Feldmann G Sievers E Frank S Strehl J Activated gammadelta T cells express the natural cytotoxicity receptor natural killer p 44 and show cytotoxic activity against myeloma cells. Clin Exp Immunol. (2006) 144:528–33. 10.1111/j.1365-2249.2006.03078.x 16734623 PMC1941970

[51] Fuchs A Cella M Kondo T Colonna M. Paradoxic inhibition of human natural interferon-producing cells by the activating receptor NKp44. Blood. (2005) 106:2076–82. 10.1182/blood-2004-12-4802 15941912

[52] Hecht M-L Rosental B Horlacher T Hershkovitz O De Paz JL Noti C. Natural cytotoxicity receptors NKp30, NKp44 and NKp46 bind to different heparan sulfate/heparin sequences. J Proteome Res. (2009) 8:712–20. 10.1021/pr800747c 19196184

[53] Mandelboim O Lieberman N Lev M Paul L Arnon TI Bushkin Y. Recognition of haemagglutinins on virus-infected cells by NKp46 activates lysis by human NK cells. Nature. (2001) 409:1055–60. 10.1038/35059110 11234016

[54] Jarahian M Fiedler M Cohnen A Djandji D Hämmerling GJ Gati C. Modulation of NKp30- and NKp46-mediated natural killer cell responses by poxviral hemagglutinin. PLoS Pathog. (2011) 7:e1002195. 10.1371/journal.ppat.1002195 21901096 PMC3161980

[55] Jarahian M Watzl C Fournier P Arnold A Djandji D Zahedi S. Activation of natural killer cells by newcastle disease virus hemagglutinin-neuraminidase. J Virol. (2009) 83:8108–21. 10.1128/JVI.00211-09 19515783 PMC2715740

[56] Arnon TI Achdout H Lieberman N Gazit R Gonen-Gross T Katz G. The mechanisms controlling the recognition of tumor- and virus-infected cells by NKp46. Blood. (2004) 103:664–72. 10.1182/blood-2003-05-1716 14504081

[57] McQuaid S Loughran S Power P Maguire P Walls D Cusi MG Haemagglutinin-neuraminidase from HPIV3 mediates human NK regulation of T cell proliferation via NKp44 and NKp46. J Gen Virol. (2018) 99:763–7. 10.1099/jgv.0.001070 29683419

[58] Mavoungou E Held J Mewono L Kremsner PG. A duffy binding-like domain is involved in the NKp30-mediated recognition of Plasmodium falciparum-parasitized erythrocytes by natural killer cells. J Infect Dis. (2007) 195:1521–31. 10.1086/515579 17436233

[59] Vankayalapati R Garg A Porgador A Griffith DE Klucar P Safi H. Role of NK cell-activating receptors and their ligands in the lysis of mononuclear phagocytes infected with an intracellular bacterium. J Immunol. (2005) 175:4611–7. 10.4049/jimmunol.175.7.4611 16177106

[60] Garg A Barnes PF Porgador A Roy S Wu S Nanda JS. Vimentin expressed on *Mycobacterium tuberculosis*-infected human monocytes is involved in binding to the NKp46 receptor. J Immunol. (2006) 177:6192–8. 10.4049/jimmunol.177.9.6192 17056548

[61] Chaushu S Wilensky A Gur C Shapira L Elboim M Halftek G. Direct recognition of Fusobacterium nucleatum by the NK cell natural cytotoxicity receptor NKp46 aggravates periodontal disease. PLoS Pathog. (2012) 8:e1002601. 10.1371/journal.ppat.1002601 22457623 PMC3310798

[62] Gur C Porgador A Elboim M Gazit R Mizrahi S Stern-Ginossar N. The activating receptor NKp46 is essential for the development of type 1 diabetes. Nat Immunol. (2010) 11:121–8. 10.1038/ni.1834 20023661

[63] Vitenshtein A Charpak-Amikam Y Yamin R Bauman Y Isaacson B Stein N. NK cell recognition of *Candida glabrata* through binding of NKp46 and NCR1 to fungal ligands Epa1, Epa6, and Epa7. Cell Host Microbe. (2016) 20:527–34. 10.1016/j.chom.2016.09.008 27736647 PMC5492882

[64] Narni-Mancinelli E Gauthier L Baratin M Guia S Fenis A Deghmane A-E. Complement factor P is a ligand for the natural killer cell-activating receptor NKp46. Sci Immunol. (2017) 2:eaam9628. 10.1126/sciimmunol.aam9628 28480349 PMC5419422

[65] Brusilovsky M Radinsky O Cohen L Yossef R Shemesh A Braiman A. Regulation of natural cytotoxicity receptors by heparan sulfate proteoglycans in -cis: a lesson from NKp44. Eur J Immunol. (2015) 45:1180–91. 10.1002/eji.201445177 25546090 PMC4415513

[66] Ho JW Hershkovitz O Peiris M Zilka A Bar-Ilan A Nal B. H5-type influenza virus hemagglutinin is functionally recognized by the natural killer-activating receptor NKp44. J Virol. (2008) 82:2028–32. 10.1128/JVI.02065-07 18077718 PMC2258730

[67] Barrow AD Edeling MA Trifonov V Luo J Goyal P Bohl B. Natural killer cells control tumor growth by sensing a growth factor. Cell. (2018) 172:534–48 e19. 10.1016/j.cell.2017.11.037 29275861 PMC6684025

[68] Gaggero S Bruschi M Petretto A Parodi M Zotto GD Lavarello C. Nidogen-1 is a novel extracellular ligand for the NKp44 activating receptor. Oncoimmunology. (2018) 7:e1470730. 10.1080/2162402X.2018.1470730 30228939 PMC6140582

[69] Rosental B Brusilovsky M Hadad U Oz D Appel MY Afergan F. Proliferating cell nuclear antigen is a novel inhibitory ligand for the natural cytotoxicity receptor NKp44. J Immunol. (2011) 187:5693–702. 10.4049/jimmunol.1102267 22021614 PMC3269963

[70] Horton NC Mathew SO Mathew PA. Novel interaction between proliferating cell nuclear antigen and HLA I on the surface of tumor cells inhibits NK cell function through NKp44. PLoS ONE. (2013) 8:e59552. 10.1371/journal.pone.0059552 23527218 PMC3602199

[71] Vieillard V Strominger JL Debré P. NK cytotoxicity against CD4+ T cells during HIV-1 infection: a gp41 peptide induces the expression of an NKp44 ligand. Proc Natl Acad Sci USA. (2005) 102:10981–6. 10.1073/pnas.0504315102 16046540 PMC1180624

[72] Baychelier F Sennepin A Ermonval M Dorgham K Debré P Vieillard V. Identification of a cellular ligand for the natural cytotoxicity receptor NKp44. Blood. (2013) 122:2935–42. 10.1182/blood-2013-03-489054 23958951

[73] Białoszewska A Baychelier F Niderla-Bielinska J Czop A Debré P Vieillard V. Constitutive expression of ligand for natural killer cell NKp44 receptor (NKp44L) by normal human articular chondrocytes. Cell Immunol. (2013) 285:6–9. 10.1016/j.cellimm.2013.08.005 24044960

[74] Hershkovitz O Rosental B Rosenberg LA Navarro-Sanchez ME Jivov S Zilka A. NKp44 receptor mediates interaction of the envelope glycoproteins from the West Nile and dengue viruses with NK cells. J Immunol. (2009) 183:2610–21. 10.4049/jimmunol.0802806 19635919 PMC2768489

[75] Esin S Batoni G Counoupas C Stringaro A Brancatisano FL Colone M. Direct binding of human NK cell natural cytotoxicity receptor NKp44 to the surfaces of mycobacteria and other bacteria. Infect Immun. (2008) 76:1719–27. 10.1128/IAI.00870-07 18212080 PMC2292874

[76] Esin S Counoupas C Aulicino A Brancatisano FL Maisetta G Bottai D. Interaction of *Mycobacterium tuberculosis* cell wall components with the human natural killer cell receptors NKp44 and Toll-like receptor 2. Scand J Immunol. (2013) 77:460–9. 10.1111/sji.12052 23578092

[77] Hershkovitz O Jarahian M Zilka A Bar-Ilan A Landau G Jivov S. Altered glycosylation of recombinant NKp30 hampers binding to heparan sulfate: a lesson for the use of recombinant immunoreceptors as an immunological tool. Glycobiology. (2008) 18:28–41. 10.1093/glycob/cwm125 18006589

[78] Warren HS Jones AL Freeman C Bettadapura J Parish CR Evidence that the cellular ligand for the human NK cell activation receptor NKp30 is not a heparan sulfate glycosaminoglycan. J Immunol. (2005) 175:207–12. 15972650 10.4049/jimmunol.175.1.207

[79] Arnon TI Achdout H Levi O Markel G Saleh N Katz G. Inhibition of the NKp30 activating receptor by pp65 of human cytomegalovirus. Nat Immunol. (2005) 6:515–23. 10.1038/ni1190 15821739

[80] Pogge von Strandmann E Simhadri VR von Tresckow B Sasse S Reiners KS Hansen HP. Human leukocyte antigen-B-associated transcript 3 is released from tumor cells and engages the NKp30 receptor on natural killer cells. Immunity. (2007) 27:965–74. 10.1016/j.immuni.2007.10.010 18055229

[81] Daßler-Plenker J Reiners KS van den Boorn JG Hansen HP Putschli B Barnert S. RIG-I activation induces the release of extracellular vesicles with antitumor activity. Oncoimmunology. (2016) 5:e1219827. 10.1080/2162402X.2016.1219827 27853642 PMC5087302

[82] Simhadri VR Reiners KS Hansen HP Topolar D Simhadri VL Nohroudi K. Dendritic cells release HLA-B-associated transcript-3 positive exosomes to regulate natural killer function. PLoS ONE. (2008) 3:e3377. 10.1371/journal.pone.0003377 18852879 PMC2566590

[83] Li Y Wang Q Mariuzza RA. Structure of the human activating natural cytotoxicity receptor NKp30 bound to its tumor cell ligand B7-H6. J Exp Med. (2011) 208:703–14. 10.1084/jem.20102548 21422170 PMC3135353

[84] Brandt CS Baratin M Yi EC Kennedy J Gao Z Fox B. The B7 family member B7-H6 is a tumor cell ligand for the activating natural killer cell receptor NKp30 in humans. J Exp Med. (2009) 206:1495–503. 10.1084/jem.20090681 19528259 PMC2715080

[85] Wang W Guo H Geng J Zheng X Wei H Sun R. Tumor-released Galectin-3, a soluble inhibitory ligand of human NKp30, plays an important role in tumor escape from NK cell attack. J Biol Chem. (2014) 289:33311–9. 10.1074/jbc.M114.603464 25315772 PMC4246088

[86] Li SS Ogbomo H Mansour MK Xiang RF Szabo L Munro F. Identification of the fungal ligand triggering cytotoxic PRR-mediated NK cell killing of *Cryptococcus* and Candida. Nat Commun. (2018) 9:751. 10.1038/s41467-018-03014-4 29467448 PMC5821813

[87] Xiang RF Li S Ogbomo H Stack D Mody CH. β1 Integrins are required to mediate NK cell killing of *Cryptococcus neoformans*. J Immunol. (2018) 201:2369–76. 10.4049/jimmunol.1701805 30201811

[88] Moretta A Bottino C Vitale M Pende D Cantoni C Mingari MC. Activating receptors and coreceptors involved in human natural killer cell-mediated cytolysis. Annu Rev Immunol. (2001) 19:197–223. 10.1146/annurev.immunol.19.1.197 11244035

[89] Joyce MG Tran P Zhuravleva MA Jaw J Colonna M Sun PD. Crystal structure of human natural cytotoxicity receptor NKp30 and identification of its ligand binding site. Proc Natl Acad Sci USA. (2011) 108:6223–8. 10.1073/pnas.1100622108 21444796 PMC3076882

[90] Schlums H Cichocki F Tesi B Theorell J Beziat V Holmes TD. Cytomegalovirus infection drives adaptive epigenetic diversification of NK cells with altered signaling and effector function. Immunity. (2015) 42:443–56. 10.1016/j.immuni.2015.02.008 25786176 PMC4612277

[91] Lee J Zhang T Hwang I Kim A Nitschke L Kim M. Epigenetic modification and antibody-dependent expansion of memory-like NK cells in human cytomegalovirus-infected individuals. Immunity. (2015) 42:431–42. 10.1016/j.immuni.2015.02.013 25786175 PMC4537797

[92] Hollyoake M Campbell RD Aguado B. NKp30 (NCR3) is a pseudogene in 12 inbred and wild mouse strains, but an expressed gene in Mus caroli. Mol Biol Evol. (2005) 22:1661–72. 10.1093/molbev/msi162 15872155

[93] Delahaye NF Rusakiewicz S Martins I Ménard C Roux S Lyonnet L. Alternatively spliced NKp30 isoforms affect the prognosis of gastrointestinal stromal tumors. Nat Med. (2011) 17:700–7. 10.1038/nm.2366 21552268

[94] Correia MP Stojanovic A Bauer K Juraeva D Tykocinski L-O Lorenz H-M. Distinct human circulating NKp30+FcεRIγ+CD8+ T cell population exhibiting high natural killer-like antitumor potential. Proc Natl Acad Sci USA. (2018) 115:E5980–9. 10.1073/pnas.1720564115 29895693 PMC6042091

[95] Halfteck GG Elboim M Gur C Achdout H Ghadially H Mandelboim O. Enhanced *in vivo* growth of lymphoma tumors in the absence of the NK-activating receptor NKp46/NCR1. J Immunol. (2009) 182:2221–30. 10.4049/jimmunol.0801878 19201876

[96] Lakshmikanth T Burke S Ali TH Kimpfler S Ursini F Ruggeri L. NCRs and DNAM-1 mediate NK cell recognition and lysis of human and mouse melanoma cell lines *in vitro* and *in vivo*. J Clin Invest. (2009) 119:1251–63. 10.1172/JCI36022 19349689 PMC2673866

[97] Glasner A Ghadially H Gur C Stanietsky N Tsukerman P Enk J. Recognition and prevention of tumor metastasis by the NK receptor NKp46/NCR1. J Immunol. (2012) 188:2509–15. 10.4049/jimmunol.1102461 22308311

[98] Merzoug LB Marie S Satoh-Takayama N Lesjean S Albanesi M Luche H. Conditional ablation of NKp46+ cells using a novel Ncr1(greenCre) mouse strain: NK cells are essential for protection against pulmonary B16 metastases. Eur J Immunol. (2014) 44:3380–91. 10.1002/eji.201444643 25142413

[99] Glasner A Isaacson B Viukov S Neuman T Friedman N Mandelboim M. Increased NK cell immunity in a transgenic mouse model of NKp46 overexpression. Sci Rep. (2017) 7:13090. 10.1038/s41598-017-12998-w 29026144 PMC5638832

[100] Glasner A Levi A Enk J Isaacson B Viukov S Orlanski S NKp46 Receptor-mediated interferon-γ production by natural killer cells increases fibronectin 1 to alter tumor architecture and control metastasis. Immunity. (2018) 48:107–19 e4. 10.1016/j.immuni.2017.12.007 29329948

[101] Cagnano E Hershkovitz O Zilka A Bar-Ilan A Golder A Sion-Vardy N. Expression of ligands to NKp46 in benign and malignant melanocytes. J Invest Dermatol. (2008) 128:972–9. 10.1038/sj.jid.5701111 17972960

[102] Hammond E Khurana A Shridhar V Dredge K. The role of heparanase and sulfatases in the modification of heparan sulfate proteoglycans within the tumor microenvironment and opportunities for novel cancer therapeutics. Front Oncol. (2014) 4:195. 10.3389/fonc.2014.00195 25105093 PMC4109498

[103] Tarbell JM Cancel LM. The glycocalyx and its significance in human medicine. J Intern Med. (2016) 280:97–113. 10.1111/joim.12465 26749537

[104] Collins LE Troeberg L. Heparan sulfate as a regulator of inflammation and immunity. J Leukoc Biol. (2019) 105:81–92. 10.1002/JLB.3RU0618-246R 30376187

[105] Raman K Kuberan B. Chemical tumor biology of heparan sulfate proteoglycans. Curr Chem Biol. (2010) 4:20–31. 10.2174/187231310790226206 20596243 PMC2892923

[106] Biroccio A Cherfils-Vicini J Augereau A Pinte S Bauwens S Ye J. TRF2 inhibits a cell-extrinsic pathway through which natural killer cells eliminate cancer cells. Nat Cell Biol. (2013) 15:818–28. 10.1038/ncb2774 23792691

[107] Textor S Bossler F Henrich K-O Gartlgruber M Pollmann J Fiegler N. The proto-oncogene Myc drives expression of the NK cell-activating NKp30 ligand B7-H6 in tumor cells. Oncoimmunology. (2016) 5:e1116674. 10.1080/2162402X.2015.1116674 27622013 PMC5007025

[108] Wang J Jin X Liu J Zhao K Xu H Wen J. The prognostic value of B7-H6 protein expression in human oral squamous cell carcinoma. J Oral Pathol Med. (2017) 46:766–72. 10.1111/jop.12586 28437013

[109] Schlecker E Fiegler N Arnold A Altevogt P Rose-John S Moldenhauer G. Metalloprotease-mediated tumor cell shedding of B7-H6, the ligand of the natural killer cell-activating receptor NKp30. Cancer Res. (2014) 74:3429–40. 10.1158/0008-5472.CAN-13-3017 24780758

[110] Mantovani S Oliviero B Lombardi A Varchetta S Mele D Sangiovanni A. Deficient natural killer cell NKp30-mediated function and altered NCR3 splice variants in hepatocellular carcinoma. Hepatology. (2018). 10.1002/hep.30235 30153337

[111] Rusakiewicz S Perier A Semeraro M Pitt JM Pogge von Strandmann E Reiners KS. NKp30 isoforms and NKp30 ligands are predictive biomarkers of response to imatinib mesylate in metastatic GIST patients. Oncoimmunology. (2017) 6:e1137418. 10.1080/2162402X.2015.1137418 28197361 PMC5283614

[112] Semeraro M Rusakiewicz S Minard-Colin V Delahaye NF Enot D Vély F. Clinical impact of the NKp30/B7-H6 axis in high-risk neuroblastoma patients. Sci Transl Med. (2015) 7:283ra55. 10.1126/scitranslmed.aaa2327 25877893

[113] Semeraro M Rusakiewicz S Zitvogel L Kroemer G. Natural killer cell mediated immunosurveillance of pediatric neuroblastoma. Oncoimmunology. (2015) 4:e1042202. 10.1080/2162402X.2015.1042202 26451315 PMC4589051

[114] Pesce S Tabellini G Cantoni C Patrizi O Coltrini D Rampinelli F. B7-H6-mediated downregulation of NKp30 in NK cells contributes to ovarian carcinoma immune escape. Oncoimmunology. (2015) 4:e1001224. 10.1080/2162402X.2014.1001224 26137398 PMC4485754

[115] Matta J Baratin M Chiche L Forel J-M Cognet C Thomas G. Induction of B7-H6, a ligand for the natural killer cell-activating receptor NKp30, in inflammatory conditions. Blood. (2013) 122:394–404. 10.1182/blood-2013-01-481705 23687088

[116] Hernandes MS Lassègue B Griendling KK. Polymerase δ-interacting Protein 2: a multifunctional protein. J Cardiovasc Pharmacol. (2017) 69:335–42. 10.1097/FJC.0000000000000465 28574953 PMC5556945

[117] Pazina T Shemesh A Brusilovsky M Porgador A Campbell KS. Regulation of the functions of natural cytotoxicity receptors by interactions with diverse ligands and alterations in splice variant expression. Front Immunol. (2017) 8:369. 10.3389/fimmu.2017.00369 28424697 PMC5371597

[118] Horton NC Mathew PA. NKp44 and natural cytotoxicity receptors as damage-associated molecular pattern recognition receptors. Front Immunol. (2015) 6:31. 10.3389/fimmu.2015.00031 25699048 PMC4313717

[119] Shemesh A Brusilovsky M Hadad U Teltsh O Edri A Rubin E. Survival in acute myeloid leukemia is associated with NKp44 splice variants. Oncotarget. (2016) 7:32933–45. 10.18632/oncotarget.8782 27102296 PMC5078064

[120] Casson J McKenna M High S. On the road to nowhere: cross-talk between post-translational protein targeting and cytosolic quality control. Biochem Soc Trans. (2016) 44:796–801. 10.1042/BST20160045 27284044

[121] Binici J Koch J. BAG-6, a jack of all trades in health and disease. Cell Mol Life Sci. (2014) 71:1829–37. 10.1007/s00018-013-1522-y 24305946 PMC11114047

[122] Reiners KS Topolar D Henke A Simhadri VR Kessler J Sauer M. Soluble ligands for NK cell receptors promote evasion of chronic lymphocytic leukemia cells from NK cell anti-tumor activity. Blood. (2013) 121:3658–65. 10.1182/blood-2013-01-476606 23509156 PMC3643764

[123] Binici J Hartmann J Herrmann J Schreiber C Beyer S Güler G. A soluble fragment of the tumor antigen BCL2-associated athanogene 6 (BAG-6) is essential and sufficient for inhibition of NKp30 receptor-dependent cytotoxicity of natural killer cells. J Biol Chem. (2013) 288:34295–303. 10.1074/jbc.M113.483602 24133212 PMC3843045

[124] Fredriksson L Li H Eriksson U. The PDGF family: four gene products form five dimeric isoforms. Cytokine Growth Factor Rev. (2004) 15:197–204. 10.1016/j.cytogfr.2004.03.007 15207811

[125] Andrae J Gallini R Betsholtz C. Role of platelet-derived growth factors in physiology and medicine. Genes Dev. (2008) 22:1276–312. 10.1101/gad.1653708 18483217 PMC2732412

[126] Glatzer T Killig M Meisig J Ommert I Luetke-Eversloh M Babic M. RORγt^+^ innate lymphoid cells acquire a proinflammatory program upon engagement of the activating receptor NKp44. Immunity. (2013) 38:1223–35. 10.1016/j.immuni.2013.05.013 23791642

[127] Ahola-Olli AV Würtz P Havulinna AS Aalto K Pitkänen N Lehtimäki T. Genome-wide association study identifies 27 Loci influencing concentrations of circulating cytokines and growth factors. Am J Hum Genet. (2017) 100:40–50. 10.1016/j.ajhg.2016.11.007 27989323 PMC5223028

[128] Ho MSP Böse K Mokkapati S Nischt R Smyth N. Nidogens-extracellular matrix linker molecules. Microsc Res Tech. (2008) 71:387–95. 10.1002/jemt.20567 18219668

[129] Li L Zhang Y Li N Feng L Yao H Zhang R. Nidogen-1: a candidate biomarker for ovarian serous cancer. Jpn J Clin Oncol. (2015) 45:176–82. 10.1093/jjco/hyu187 25378651

[130] Willumsen N Bager CL Leeming DJ Bay-Jensen A-C Karsdal MA. Nidogen-1 degraded by Cathepsin S can be quantified in serum and is associated with non-small cell lung cancer. Neoplasia. (2017) 19:271–8. 10.1016/j.neo.2017.01.008 28282545 PMC5344320

[131] Farhad M Rolig AS Redmond WL. The role of Galectin-3 in modulating tumor growth and immunosuppression within the tumor microenvironment. Oncoimmunology. (2018) 7:e1434467. 10.1080/2162402X.2018.1434467 29872573 PMC5980349

[132] Spits H Artis D Colonna M Diefenbach A Di Santo JP Eberl G. Innate lymphoid cells–a proposal for uniform nomenclature. Nat Rev Immunol. (2013) 13:145–9. 10.1038/nri3365 23348417

[133] Barrow AD Colonna M. Innate lymphoid cell sensing of tissue vitality. Curr Opin Immunol. (2018) 56:82–93. 10.1016/j.coi.2018.11.004 30529190 PMC6469350

[134] Cortez VS Cervantes-Barragan L Robinette ML Bando JK Wang Y Geiger TL. Transforming growth factor-β signaling guides the differentiation of innate lymphoid cells in salivary glands. Immunity. (2016) 44:1127–39. 10.1016/j.immuni.2016.03.007 27156386 PMC5114145

[135] Smyth MJ Cretney E Takeda K Wiltrout RH Sedger LM Kayagaki N. Tumor necrosis factor–related apoptosis-inducing ligand (Trail) contributes to interferon γ-dependent natural killer cell protection from tumor metastasis. J Exp Med. (2001) 193:661–70. 10.1084/jem.193.6.661 11257133 PMC2193421

[136] Stegmann KA Björkström NK Veber H Ciesek S Riese P Wiegand J. Interferon-alpha-induced TRAIL on natural killer cells is associated with control of hepatitis C virus infection. Gastroenterology. (2010) 138:1885–97. 10.1053/j.gastro.2010.01.051 20334827

[137] Kayagaki N Yamaguchi N Nakayama M Takeda K Akiba H Tsutsui H. Expression and function of TNF-related apoptosis-inducing ligand on murine activated NK cells. J Immunol. (1999) 163:1906–13. 10438925

[138] Takeda K Smyth MJ Cretney E Hayakawa Y Yamaguchi N Yagita H. Involvement of tumor necrosis factor-related apoptosis-inducing ligand in NK cell-mediated and IFN-gamma-dependent suppression of subcutaneous tumor growth. Cell Immunol. (2001) 214:194–200. 10.1006/cimm.2001.1896 12088418

[139] Cretney E Takeda K Yagita H Glaccum M Peschon JJ Smyth MJ. Increased susceptibility to tumor initiation and metastasis in TNF-related apoptosis-inducing ligand-deficient mice. J Immunol. (2002) 168:1356–61. 10.4049/jimmunol.168.3.1356 11801676

[140] Seki N Hayakawa Y Brooks AD Wine J Wiltrout RH Yagita H. Tumor necrosis factor-related apoptosis-inducing ligand-mediated apoptosis is an important endogenous mechanism for resistance to liver metastases in murine renal cancer. Cancer Res. (2003) 63:207–13. 12517799

[141] Zerafa N Westwood JA Cretney E Mitchell S Waring P Iezzi M. Cutting edge: TRAIL deficiency accelerates hematological malignancies. J Immunol. (2005) 175:5586–90. 10.4049/jimmunol.175.9.5586 16237043

[142] Sheppard S Schuster IS Andoniou CE Cocita C Adejumo T Kung SKP. The murine natural cytotoxic receptor NKp46/NCR1 controls TRAIL protein expression in NK cells and ILC1s. Cell Rep. (2018) 22:3385–92. 10.1016/j.celrep.2018.03.023 29590608 PMC5896200

[143] Turchinovich G Ganter S Bärenwaldt A Finke D. NKp46 calibrates tumoricidal potential of type 1 innate lymphocytes by regulating TRAIL expression. J Immunol. (2018) 200:3762–8. 10.4049/jimmunol.1701333 29661825

[144] Schuster IS Wikstrom ME Brizard G Coudert JD Estcourt MJ Manzur M. TRAIL+ NK cells control CD4+ T cell responses during chronic viral infection to limit autoimmunity. Immunity. (2014) 41:646–56. 10.1016/j.immuni.2014.09.013 25367576

[145] Dunn C Brunetto M Reynolds G Christophides T Kennedy PT Lampertico P. Cytokines induced during chronic hepatitis B virus infection promote a pathway for NK cell-mediated liver damage. J Exp Med. (2007) 204:667–80. 10.1084/jem.20061287 17353365 PMC2137916

[146] Peppa D Gill US Reynolds G Easom NJW Pallett LJ Schurich A. Up-regulation of a death receptor renders antiviral T cells susceptible to NK cell-mediated deletion. J Exp Med. (2013) 210:99–114. 10.1084/jem.20121172 23254287 PMC3549717

[147] Almeida AR Correia DV Fernandes-Platzgummer A da Silva CL da Silva MG Anjos DR. Delta one T cells for immunotherapy of chronic lymphocytic leukemia: clinical-grade expansion/differentiation and preclinical proof of concept. Clin Cancer Res. (2016) 22:5795–804. 10.1158/1078-0432.CCR-16-0597 27307596

[148] Crome SQ Nguyen LT Lopez-Verges S Yang SYC Martin B Yam JY. A distinct innate lymphoid cell population regulates tumor-associated T cells. Nat Med. (2017) 23:368–75. 10.1038/nm.4278 28165478 PMC5497996

[149] Neill DR Wong SH Bellosi A Flynn RJ Daly M Langford TKA. Nuocytes represent a new innate effector leukocyte that mediates type-2 immunity. Nature. (2010) 464:1367–70. 10.1038/nature08900 20200518 PMC2862165

[150] Klose CSN Artis D. Innate lymphoid cells as regulators of immunity, inflammation and tissue homeostasis. Nat Immunol. (2016) 17:765–74. 10.1038/ni.3489 27328006

[151] Xue L Salimi M Panse I Mjösberg JM McKenzie ANJ Spits H. Prostaglandin D2 activates group 2 innate lymphoid cells through chemoattractant receptor-homologous molecule expressed on TH2 cells. J Allergy Clin Immunol. (2014) 133:1184–94. 10.1016/j.jaci.2013.10.056 24388011 PMC3979107

[152] Trabanelli S Chevalier MF Martinez-Usatorre A Gomez-Cadena A Salomé B Lecciso M. Tumour-derived PGD2 and NKp30-B7H6 engagement drives an immunosuppressive ILC2-MDSC axis. Nat Commun. (2017) 8:593. 10.1038/s41467-017-00678-2 28928446 PMC5605498

[153] Diab M Glasner A Isaacson B Bar-On Y Drori Y Yamin R. NK-cell receptors NKp46 and NCR1 control human metapneumovirus infection. Eur J Immunol. (2017) 47:692–703. 10.1002/eji.201646756 28191644

[154] Miletic A Lenartic M Popovic B Brizic I Trsan T Miklic K. NCR1-deficiency diminishes the generation of protective murine cytomegalovirus antibodies by limiting follicular helper T-cell maturation. Eur J Immunol. (2017) 47:1443–56. 10.1002/eji.201646763 28643847

[155] Charpak-Amikam Y Kubsch T Seidel E Oiknine-Djian E Cavaletto N Yamin R. Human cytomegalovirus escapes immune recognition by NK cells through the downregulation of B7-H6 by the viral genes US18 and US20. Sci Rep. (2017) 7:8661. 10.1038/s41598-017-08866-2 28819195 PMC5561058

[156] Schmiedel D Tai J Levi-Schaffer F Dovrat S Mandelboim O. Human Herpesvirus 6B downregulates expression of activating ligands during lytic infection to escape elimination by natural killer cells. J Virol. (2016) 90:9608–17. 10.1128/JVI.01164-16 27535049 PMC5068514

[157] Liang Y Song D-Z Liang S Zhang Z-F Gao L-X Fan X-H. The hemagglutinin-neuramidinase protein of Newcastle disease virus upregulates expression of the TRAIL gene in murine natural killer cells through the activation of Syk and NF-κB. PLoS ONE. (2017) 12:e0178746. 10.1371/journal.pone.0178746 28614370 PMC5470681

[158] Gazit R Gruda R Elboim M Arnon TI Katz G Achdout H. Lethal influenza infection in the absence of the natural killer cell receptor gene Ncr1. Nat Immunol. (2006) 7:517–23. 10.1038/ni1322 16565719

[159] Jang Y Gerbec ZJ Won T Choi B Podsiad A B Moore B. Cutting edge: check your mice-a point mutation in the Ncr1 locus identified in CD45.1 congenic mice with consequences in mouse susceptibility to infection. J Immunol. (2018) 200:1982–7. 10.4049/jimmunol.1701676 29440507 PMC5840015

[160] Mao H Tu W Liu Y Qin G Zheng J Chan P-L. Inhibition of human natural killer cell activity by influenza virions and hemagglutinin. J Virol. (2010) 84:4148–57. 10.1128/JVI.02340-09 20164232 PMC2863726

[161] Bar-On Y Seidel E Tsukerman P Mandelboim M Mandelboim O. Influenza virus uses its neuraminidase protein to evade the recognition of two activating NK cell receptors. J Infect Dis. (2014) 210:410–8. 10.1093/infdis/jiu094 24532603 PMC4074429

[162] Divaris K Monda KL North KE Olshan AF Reynolds LM Hsueh W-C. Exploring the genetic basis of chronic periodontitis: a genome-wide association study. Hum Mol Genet. (2013) 22:2312–24. 10.1093/hmg/ddt065 23459936 PMC3652417

[163] Augugliaro R Parolini S Castriconi R Marcenaro E Cantoni C Nanni M. Selective cross-talk among natural cytotoxicity receptors in human natural killer cells. Eur J Immunol. (2003) 33:1235–41. 10.1002/eji.200323896 12731048

[164] Li SS Kyei SK Timm-McCann M Ogbomo H Jones GJ Shi M. The NK receptor NKp30 mediates direct fungal recognition and killing and is diminished in NK cells from HIV-infected patients. Cell Host Microbe. (2013) 14:387–97. 10.1016/j.chom.2013.09.007 24139398

[165] Kemper C Atkinson JP Hourcade DE. Properdin: emerging roles of a pattern-recognition molecule. Annu Rev Immunol. (2010) 28:131–55. 10.1146/annurev-immunol-030409-101250 19947883

[166] Sprong T Roos D Weemaes C Neeleman C Geesing CLM Mollnes TE. Deficient alternative complement pathway activation due to factor D deficiency by 2 novel mutations in the complement factor D gene in a family with meningococcal infections. Blood. (2006) 107:4865–70. 10.1182/blood-2005-07-2820 16527897

[167] Hudspeth K Fogli M Correia DV Mikulak J Roberto A Della Bella S. Engagement of NKp30 on Vδ1 T cells induces the production of CCL3, CCL4, and CCL5 and suppresses HIV-1 replication. Blood. (2012) 119:4013–6. 10.1182/blood-2011-11-390153 22403253

[168] Wilkens J Male V Ghazal P Forster T Gibson DA Williams ARW. Uterine NK cells regulate endometrial bleeding in women and are suppressed by the progesterone receptor modulator asoprisnil. J Immunol. (2013) 191:2226–35. 10.4049/jimmunol.1300958 23913972 PMC3843142

[169] Moffett A Colucci F. Uterine NK cells: active regulators at the maternal-fetal interface. J Clin Invest. (2014) 124:1872–9. 10.1172/JCI68107 24789879 PMC4001528

[170] Filipovic I Chiossone L Vacca P Hamilton RS Ingegnere T Doisne J-M. Molecular definition of group 1 innate lymphoid cells in the mouse uterus. Nat Commun. (2018) 9:4492. 10.1038/s41467-018-06918-3 30374017 PMC6206068

[171] Hanna J Goldman-Wohl D Hamani Y Avraham I Greenfield C Natanson-Yaron S. Decidual NK cells regulate key developmental processes at the human fetal-maternal interface. Nat Med. (2006) 12:1065–74. 10.1038/nm1452 16892062

[172] El Costa H Casemayou A Aguerre-Girr M Rabot M Berrebi A Parant O. Critical and differential roles of NKp46- and NKp30-activating receptors expressed by uterine NK cells in early pregnancy. J Immunol. (2008) 181:3009–17. 10.4049/jimmunol.181.5.3009 18713971

[173] El Costa H Tabiasco J Berrebi A Parant O Aguerre-Girr M Piccinni M-P. Effector functions of human decidual NK cells in healthy early pregnancy are dependent on the specific engagement of natural cytotoxicity receptors. J Reprod Immunol. (2009) 82:142–7. 10.1016/j.jri.2009.06.123 19615756

[174] Siewiera J Gouilly J Hocine H-R Cartron G Levy C Al-Daccak R. Natural cytotoxicity receptor splice variants orchestrate the distinct functions of human natural killer cell subtypes. Nat Commun. (2015) 6:10183. 10.1038/ncomms10183 26666685 PMC4682172

[175] Carbone T Nasorri F Pennino D Eyerich K Foerster S Cifaldi L. CD56highCD16-CD62L- NK cells accumulate in allergic contact dermatitis and contribute to the expression of allergic responses. J Immunol. (2010) 184:1102–10. 10.4049/jimmunol.0902518 20008290

[176] O'Leary JG Goodarzi M Drayton DL von Andrian UH. T cell- and B cell-independent adaptive immunity mediated by natural killer cells. Nat Immunol. (2006) 7:507–16. 10.1038/ni1332 16617337

[177] Rouzaire P Luci C Blasco E Bienvenu J Walzer T Nicolas J-F. Natural killer cells and T cells induce different types of skin reactions during recall responses to haptens. Eur J Immunol. (2012) 42:80–8. 10.1002/eji.201141820 21968602

[178] Ghadially H Horani A Glasner A Elboim M Gazit R Shoseyov D. NKp46 regulates allergic responses. Eur J Immunol. (2013) 43:3006–16. 10.1002/eji.201343388 23878025 PMC3867659

[179] Elhaik Goldman S Moshkovits I Shemesh A Filiba A Tsirulsky Y Vronov E. Natural killer receptor 1 dampens the development of allergic eosinophilic airway inflammation. PLoS ONE. (2016) 11:e0160779. 10.1371/journal.pone.0160779 27580126 PMC5007051

[180] Salimi M Xue L Jolin H Hardman C Cousins DJ McKenzie ANJ. Group 2 innate lymphoid cells express functional NKp30 receptor inducing type 2 cytokine production. J Immunol. (2016) 196:45–54. 10.4049/jimmunol.1501102 26582946 PMC4913864

[181] Poirot L Benoist C Mathis D. Natural killer cells distinguish innocuous and destructive forms of pancreatic islet autoimmunity. Proc Natl Acad Sci USA. (2004) 101:8102–7. 10.1073/pnas.0402065101 15141080 PMC419564

[182] Alba A Planas R Clemente X Carrillo J Ampudia R Puertas M-C. Natural killer cells are required for accelerated type 1 diabetes driven by interferon-beta. Clin Exp Immunol. (2008) 151:467–75. 10.1111/j.1365-2249.2007.03580.x 18190608 PMC2276969

[183] Traherne JA Jiang W Valdes AM Hollenbach JA Jayaraman J Lane JA. KIR haplotypes are associated with late-onset type 1 diabetes in European-American families. Genes Immun. (2016) 17:8–12. 10.1038/gene.2015.44 26492518 PMC4746488

[184] Wang Y Yuan W Guo H Jiang Y. High frequency of activated NKp46(+) natural killer cells in patients with new diagnosed of latent autoimmune diabetes in adults. Autoimmunity. (2015) 48:267–73. 10.3109/08916934.2014.990629 25495606

[185] Yossef R Gur C Shemesh A Guttman O Hadad U Nedvetzki S. Targeting natural killer cell reactivity by employing antibody to NKp46: implications for type 1 diabetes. PLoS ONE. (2015) 10:e0118936. 10.1371/journal.pone.0118936 25719382 PMC4342013

[186] Berhani O Glasner A Kahlon S Duev-Cohen A Yamin R Horwitz E. Human anti-NKp46 antibody for studies of NKp46-dependent NK cell function and its applications for type 1 diabetes and cancer research. Eur J Immunol. (2018) 10.1002/eji.201847611 30536875

[187] Rusakiewicz S Nocturne G Lazure T Semeraro M Flament C Caillat-Zucman S. NCR3/NKp30 contributes to pathogenesis in primary Sjogren's syndrome. Sci Transl Med. (2013) 5:195ra96. 10.1126/scitranslmed.3005727 23884468 PMC4237161

[188] Chappell JD Prota AE Dermody TS Stehle T. Crystal structure of reovirus attachment protein sigma1 reveals evolutionary relationship to adenovirus fiber. EMBO J. (2002) 21:1–11. 10.1093/emboj/21.1.1 11782420 PMC125343

[189] Bar-On Y Charpak-Amikam Y Glasner A Isaacson B Duev-Cohen A Tsukerman P. NKp46 Recognizes the sigma1 protein of reovirus: implications for reovirus-based cancer therapy. J Virol. (2017) 91:e01045–17. 10.1128/JVI.01045-17 28724773 PMC5599737

[190] Wu M-R Zhang T Gacerez AT Coupet TA DeMars LR Sentman CL. B7H6-specific bispecific T cell engagers lead to tumor elimination and host antitumor immunity. J Immunol. (2015) 194:5305–11. 10.4049/jimmunol.1402517 25911747 PMC4433849

[191] Peipp M Derer S Lohse S Staudinger M Klausz K Valerius T. HER2-specific immunoligands engaging NKp30 or NKp80 trigger NK-cell-mediated lysis of tumor cells and enhance antibody-dependent cell-mediated cytotoxicity. Oncotarget. (2015) 6:32075–88. 10.18632/oncotarget.5135 26392331 PMC4741660

[192] Kellner C Maurer T Hallack D Repp R van de Winkel JGJ Parren PWHI. Mimicking an induced self phenotype by coating lymphomas with the NKp30 ligand B7-H6 promotes NK cell cytotoxicity. J Immunol. (2012) 189:5037–46. 10.4049/jimmunol.1201321 23066150

[193] Kellner C Günther A Humpe A Repp R Klausz K Derer S Enhancing natural killer cell-mediated lysis of lymphoma cells by combining therapeutic antibodies with CD20-specific immunoligands engaging NKG2D or NKp30. Oncoimmunology. (2016) 5:e1058459 10.1080/2162402X.2015.1058459 26942070 PMC4760288

[194] Zhang T Wu M-R Sentman CL. An NKp30-based chimeric antigen receptor promotes T cell effector functions and antitumor efficacy *in vivo*. J Immunol. (2012) 189:2290–9. 10.4049/jimmunol.1103495 22851709 PMC3633481

[195] Wu M-R Zhang T DeMars LR Sentman CL. B7H6-specific chimeric antigen receptors lead to tumor elimination and host antitumor immunity. Gene Ther. (2015) 22:675–84. 10.1038/gt.2015.29 25830550 PMC4529373

[196] Hua CK Gacerez AT Sentman CL Ackerman ME. Development of unique cytotoxic chimeric antigen receptors based on human scFv targeting B7H6. Protein Eng Des Sel. (2017) 30:713–21. 10.1093/protein/gzx051 29040754 PMC5914360

[197] Tal Y Yaakobi S Horovitz-Fried M Safyon E Rosental B Porgador A. An NCR1-based chimeric receptor endows T-cells with multiple anti-tumor specificities. Oncotarget. (2014) 5:10949–58. 10.18632/oncotarget.1919 25431955 PMC4279421

[198] Eisenberg V Shamalov K Meir S Hoogi S Sarkar R Pinker S. Targeting multiple tumors using T-cells engineered to express a natural cytotoxicity receptor 2-based chimeric receptor. Front Immunol. (2017) 8:1212. 10.3389/fimmu.2017.01212 29085357 PMC5649149

[199] Cerwenka A Bakker AB McClanahan T Wagner J Wu J Phillips JH. Retinoic acid early inducible genes define a ligand family for the activating NKG2D receptor in mice. Immunity. (2000) 12:721–7. 10.1016/S1074-7613(00)80222-8 10894171

[200] Barrow AD Trowsdale J. The extended human leukocyte receptor complex: diverse ways of modulating immune responses. Immunol Rev. (2008) 224:98–123. 10.1111/j.1600-065X.2008.00653.x 18759923

[201] Barrow AD Raynal N Andersen TL Slatter DA Bihan D Pugh N. OSCAR is a collagen receptor that costimulates osteoclastogenesis in DAP12-deficient humans and mice. J Clin Invest. (2011) 121:3505–16. 10.1172/JCI45913 21841309 PMC3163954

[202] Zhou L Hinerman JM Blaszczyk M Miller JLC Conrady DG Barrow AD. Structural basis for collagen recognition by the immune receptor OSCAR. Blood. (2016) 127:529–37. 10.1182/blood-2015-08-667055 26552697 PMC4742545

[203] Haywood J Qi J Chen C-C Lu G Liu Y Yan J. Structural basis of collagen recognition by human osteoclast-associated receptor and design of osteoclastogenesis inhibitors. Proc Natl Acad Sci USA. (2016) 113:1038–43. 10.1073/pnas.1522572113 26744311 PMC4743793

[204] Barrow AD Palarasah Y Bugatti M Holehouse AS Byers DE Holtzman MJ. OSCAR is a receptor for surfactant protein D that activates TNF-α release from human CCR2+ inflammatory monocytes. J Immunol. (2015) 194:3317–26. 10.4049/jimmunol.1402289 25716998 PMC4369396

[205] Brondijk THC de Ruiter T Ballering J Wienk H Lebbink RJ van Ingen H. Crystal structure and collagen-binding site of immune inhibitory receptor LAIR-1: unexpected implications for collagen binding by platelet receptor GPVI. Blood. (2010) 115:1364–73. 10.1182/blood-2009-10-246322 20007810

[206] Lebbink RJ Raynal N de Ruiter T Bihan DG Farndale RW Meyaard L. Identification of multiple potent binding sites for human leukocyte associated Ig-like receptor LAIR on collagens II and III. Matrix Biol. (2009) 28:202–10. 10.1016/j.matbio.2009.03.005 19345263

[207] Olde Nordkamp MJM van Eijk M Urbanus RT Bont L Haagsman HP Meyaard L. Leukocyte-associated Ig-like receptor-1 is a novel inhibitory receptor for surfactant protein D. J Leukoc Biol. (2014) 96:105–11. 10.1189/jlb.3AB0213-092RR 24585933

[208] Bauer S Groh V Wu J Steinle A Phillips JH Lanier LL. Activation of NK cells and T cells by NKG2D, a receptor for stress-inducible MICA. Science. (1999) 285:727–9. 10426993 10.1126/science.285.5428.727

[209] Eagle RA Trowsdale J. Promiscuity and the single receptor: NKG2D. Nat Rev Immunol. (2007) 7:737–44. 10.1038/nri2144 17673918

[210] Deng W Gowen BG Zhang L Wang L Lau S Iannello A. Antitumor immunity. A shed NKG2D ligand that promotes natural killer cell activation and tumor rejection. Science. (2015) 348:136–9. 10.1126/science.1258867 25745066 PMC4856222

[211] Eagle RA Flack G Warford A Martínez-Borra J Jafferji I Traherne JA. Cellular expression, trafficking, and function of two isoforms of human ULBP5/RAET1G. PLoS ONE. (2009) 4:e4503. 10.1371/journal.pone.0004503 19223974 PMC2637608

[212] Barrow AD Colonna M. Tailoring natural killer cell immunotherapy to the tumour microenvironment. Semin Immunol. (2017) 31:30–6. 10.1016/j.smim.2017.09.001 28935344 PMC5659759

[213] Barrow AD Colonna M. Exploiting NK cell surveillance pathways for cancer therapy. Cancers. (2019) 11:55. 10.3390/cancers11010055 30626155 PMC6356551

[214] Chang ZL Lorenzini MH Chen X Tran U Bangayan NJ Chen YY. Rewiring T-cell responses to soluble factors with chimeric antigen receptors. Nat Chem Biol. (2018) 14:317–24. 10.1038/nchembio.2565 29377003 PMC6035732

[215] Hou AJ Chang ZL Lorenzini MH Zah E Chen YY. TGF-β-responsive CAR-T cells promote anti-tumor immune function. Bioeng Transl Med. (2018) 3:75–86. 10.1002/btm2.10097 30065964 PMC6063867

